# Screening for candidate genes related with histological microstructure, meat quality and carcass characteristic in pig based on RNA-seq data

**DOI:** 10.5713/ajas.17.0714

**Published:** 2018-03-13

**Authors:** Katarzyna Ropka-Molik, Anna Bereta, Kacper Żukowski, Mirosław Tyra, Katarzyna Piórkowska, Grzegorz Żak, Maria Oczkowicz

**Affiliations:** 1 Department of Genomics and Animal Molecular Biology, National Research Institute of Animal Production, Krakowska 1, Balice 32-083, Poland; 2 Department of Animal Genetics and Breeding, National Research Institute of Animal Production, Krakowska 1, Balice 32-083, Poland

**Keywords:** QTLs, Candidate Genes, Polymorphism, Histological Traits, Fibers Types

## Abstract

**Objective:**

The aim of the present study was to identify genetic variants based on RNA-seq data, obtained via transcriptome sequencing of muscle tissue of pigs differing in muscle histological structure, and to verify the variants’ effect on histological microstructure and production traits in a larger pig population.

**Methods:**

RNA-seq data was used to identify the panel of single nucleotide polymorphisms (SNPs) significantly related with percentage and diameter of each fiber type (I, IIA, IIB). Detected polymorphisms were mapped to quantitative trait loci (QTLs) regions. Next, the association study was performed on 944 animals representing five breeds (Landrace, Large White, Pietrain, Duroc, and native Puławska breed) in order to evaluate the relationship of selected SNPs and histological characteristics, meat quality and carcasses traits.

**Results:**

Mapping of detected genetic variants to QTL regions showed that chromosome 14 was the most overrepresented with the identification of four QTLs related to percentage of fiber types I and IIA. The association study performed on a 293 *longissimus* muscle samples confirmed a significant positive effect of transforming acidic coiled-coil-containing protein 2 (*TACC2*) polymorphisms on fiber diameter, while SNP within forkhead box O1 (*FOXO1*) locus was associated with decrease of diameter of fiber types IIA and IIB. Moreover, subsequent general linear model analysis showed significant relationship of *FOXO1*, delta 4-desaturase, sphingolipid 1 (*DEGS1*), and troponin T2 (*TNNT2*) genes with loin ‘eye’ area, *FOXO1* with loin weight, as well as *FOXO1* and *TACC2* with lean meat percentage. Furthermore, the intramuscular fat content was positively associated (p<0.01) with occurrence of polymorphisms within *DEGS1*, *TNNT2* genes and negatively with occurrence of *TACC2* polymorphism.

**Conclusion:**

This study’s results indicate that the SNP calling analysis based on RNA-seq data can be used to search candidate genes and establish the genetic basis of phenotypic traits. The presented results can be used for future studies evaluating the use of selected SNPs as genetic markers related to muscle histological profile and production traits in pig breeding.

## INTRODUCTION

Analyzing transcriptomes using next generation sequencing methods (RNA-seq) has been useful in various applications: estimation of gene expression; identification of novel genes or transcripts; and detection of genomic variants, gene fusion and RNA editing [1,2]. According to numerous studies, calling variants from RNA-seq data has many advantages. First, RNA sequencing is less cost-intensive compared to genome sequencing, and often, genomic variants can be identified from the existing RNA-seq data [3]. Furthermore, the comparison of single nucleotide polymorphism (SNP) panels obtained from animals differing in the levels of given traits can be useful in searching for the genetic background of analyzed traits. To date, the most efficient and reliable methods of SNP calling from RNA-seq data have been validated [4,5].

In pigs, an RNA-seq approach enabled Jung et al [6] to detect nonsynonymous single nucleotide variations potentially related to enzyme activity in porcine liver. Moreover, the authors showed that selected SNPs were associated with pork quality and could be used in marker-assisted selection in future pig breeding programs. Transcriptome sequencing of liver tissue collected from four different pig breeds enabled the identification of non-synonymous SNPs within genes coding for enzymes that are critical during post-mortem metabolic process in muscle [7]. Using RNA-seq data, Piórkowska et al [8] compared distributions of genetic variants detected in two breeds with extreme fatness parameters and identified genes involved in fatty acid metabolism and lipid storage, which can be potential genetic markers associated with meat quality in pigs. Conversely, Fisher et al [9] detected non-synonymous mutations with a possible influence on porcine reproduction traits using transcriptome data obtained after RNA sequence from oviduct and testis tissue.

It has been well-established that percentage number of fiber types in muscle determines metabolic properties and water holding capacity of muscle tissue, as well as marbling, intramuscular fat (IMF) level, meat color and texture [10–12]. In our previous study performed on Large White pigs, we identified genes differentially expressed in muscle contingent on histological microstructure [13]. According to the differential expression and biological function, several genes were selected with possible effect on variation of fiber-type distribution. Thus, the aim of the present study was to identify genetic variants based on RNA-seq data, obtained via transcriptome sequencing of muscle tissue of pigs differing in muscle histological structure. The second aim was to select the most interesting variants, based on transcriptome-wide association study, and to verify the theirs effect on production traits and histological microstructure in a larger pig population.

## MATERIALS AND METHODS

### RNA-seq data

The SNPs in coding and untranslated regions (UTR) were identified based on RNA-seq data obtained previously by Ropka-Molik et al [13]. Whole transcriptome sequencing was performed on *longissimus lumborum* samples collected from 11 Large White pigs that differed in the histological structure of this muscle. Pigs (unrelated sows) were maintained under the same housing and feeding conditions and slaughtered at final body weight of 100 kg (±2.5 kg). Immediately after slaughter, muscle samples were collected and stored at −20°C in RNAlater solution (Ambion Inc., Austin, TX, USA) for RNA-seq application and at −80°C for histological analysis. The details of the animals used, the RNA-seq experiment and histological analysis were described in a previous study focused on differential expression analysis [13].

### SNPs identification and association study based on RNA-seq data

Quality control of the raw RNA-seq data was performed using FastQC (v0.11.4; Illumina, San Diego, CA, USA). Adapters, poly-A sequences, and reads with a quality score lower than 20 and a fragment size reads lower than 36 bp were removed using the Flexbar tool (v.2.5GNU General Public License version 3.0 [GPLv3]). The cleaned reads were mapped to a *Sus scrofa* reference genome (Sscrofa10.2) using STAR aligner [14]. Next, reads containing Ns (N cigar elements) were split (CIGAR string), and base quality score recalibration, INDEL realignment, duplicate removal, and identification of SNPs and INDELs (small insertions and deletions) were performed using Picard and GATK software packages [15]. The filtering parameters were as follows: FisherStrand value>50, QualByDepth<2.0, RMS mapping quality<40, quality<40 and a SnpCluster filter. The genome annotation of identified polymorphisms was established using SnpEff 4.1b and Variant Effect Predictor (EMBL-European Bioinformatics Institute, Hinxton, Cambridge, UK) [16], and for downstream association analysis, only SNPs located within coding regions were selected (5′prime UTR variants; missense variants; synonymous variants; 3′prime UTR variants; noncoding transcript exon variants).

The association analysis for additive (fixed) effect of SNP was carried out for percentage and diameter of the I, IIA, and IIB types of fibers. The whole procedure was implemented in R-project. SNPs were considered significant if they met the following criteria: frequency (chisq p<0.05), a minimum of 9 observations, and a significant association analysis (p<0.05).

### QTLs identification

The differential expressed genes (DEGs) identified by RNA-seq, and genes with SNPs (based on whole coding sequence analysis) significantly associated with percentage and/or diameter of I, IIA, and IIB types of fibers were mapped to quantitative trait loci (QTL) regions. The QTLs positions were taken from Animal Quantitative Trait Loci Database (Animal QTLdb) [17], which corresponds to the genome release used in this study (Sscrofta10.2, Ensemble database). We used R script [18] prepared by us to conduit the whole procedure. Based on RNA-seq results, 355 DEGs were mapped to QTLs regions. In contrast, based on the transcriptome-wide association study, 222 polymorphisms correlated with at least two histological traits and located within 149 genes were selected to the subsequent analysis.

### Candidate genes – association study on a larger population

For an association study, four genes were selected based on high gene expression differences during RNA-seq analysis and their location within QTLs related to percentage of fibers I, IIA, and IIB (troponin T2 [*TNNT2*]; forkhead box O1 [*FOXO1*]). Two additional polymorphisms within two genes related to analyzed histological properties (transforming acidic coiled-coil-containing protein 2 [*TACC2*]; delta 4-desaturase, sphingolipid 1 [*DEGS1*]) were selected as significant based on the transcriptome-wide association study. For all genes, genotypes were assessed using polymerase chain reaction-restriction fragment length polymorphism (PCR-RFLP) method (*TNNT2*; *TACC2*; *DEGS1*) and polymerase chain reaction-artificially created restriction site (PCR-ACRS) method (*FOXO1*). The details of the analyses were presented in Table 1.

For the downstream association study, a larger pig population comprising 944 animals representing five breeds (Landrace n = 306, Large White n = 345, Pietrain n = 80, Duroc n = 61, native Puławska breed n = 152) was used. All pigs (sows) were unrelated at least three generations back and were maintained under the same housing and feeding conditions according to Pig Test Station procedures. In the Test Station pigs are slaughtered, dissected and after carcass evaluation, meat is standard intended for consumption, thus research does not require the approval of Animal Experimentation committee. Pigs were fattened from 30 kg up to 100 (±2.5) kg according to test procedures described previously by Ropka-Molik et al [19] and were slaughtered and dissected. For all animals, the following carcass characteristic traits were measured: weight of loin (kg), loin “eye” area (cm) and lean meat percentage (%). Furthermore, several meat quality traits were measured for selected animals: IMF content (the Soxhlet method) [20] and meat color (redness, a*; yellowness, b*; and lightness, L* parameters; Minolta Chroma Meter CR-310; Osaka, Japan) (699 pigs). The determination of meat color was performed using Chroma Meter CR-310 with a pulse xenon arc lamp D65 and 50 mm orifice. The a*, b*, and L* parameters were assessed 24 h *post mortem* on muscle tissue samples (*longissimus lumborum*; 150 to 200 g). The analysis was performed in two measurement points for each sample.

For selected animals, in total 293 (Landrace n = 110, Large White n = 70, Duroc n = 20, Puławska pigs n = 93), *longissimus* muscle samples were collected immediately after slaughter and histological profile—percentage and diameter of the I, IIA, and IIB types of fibers—were estimated according to the procedure described previously by Ropka-Molik et al [13]. The animals used to histological analysis were randomly selected from population included in association study. Tissue samples (frozen at −80°C) were cut on 10 μm thick slides using a cryostat (Slee MEV, Mainz, Germany) and then fibers were detected using NADPH dehydrogenase (diaphorase) method. Myosin heavy chains (MHCs) were detected immunohistochemically with NCL-MHC antibodies (Leica, Novocasta, Leider Lane, Buffalo Grove, IL, USA) and NovoLink Polymer Detection Systems (Zalesie Gorne, Poland) (Leica, Novocasta, USA) [21]. The obtained preparations were analyzed in terms of the percentage content of types of muscle fibers (I, IIA, IIB) and their diameter using the Nikon Eclipse 50i light microscope and MultiScan image analysis software (ver. 18.03; Sony, Tokyo, Japan). In each histological sample, the analysis covered a minimum of 300 fibers of each types. The percentage of fiber types was calculated based on 10 bundles measurements.

The statistical analysis for all selected genes was performed using the general linear model (GLM) procedure in SAS software (SASv. 8.02; Wadowice, Poland) and the model used was:

$${\text{Y}}_{\text{ijk}}=\mu +{\text{f}}_{\text{i}}+{\text{h}}_{\text{j}}+\alpha ({\text{x}}_{\text{ij}})+{\text{e}}_{\text{ijk}}$$

Where, Y_ijk_, the trait measured; μ, the overall mean for the trait; f_i_, the fixed effect of j genotype group of analyzed gene; h_j_, the fixed effect of k breed; α(x_ij_), covariate with weight of right half-carcass for selected slaughter traits; e_ijk_, random error.

The correlation coefficients between histological features and carcass characteristics and meat quality traits were calculated using Pearson correlation method (SAS software, SASv. 8.02, Poland).

## RESULTS

### SNP identification and transcriptome-wide association analysis

Based on RNA-seq data, 65,535 SNPs were identified. After filtering according to the criteria outlined above, 51,590 SNPs were selected for use in the association study. The significant association of SNPs and percentage of I, IIA, and IIB fiber types were identified (p<0.05; 560, 251, and 837; respectively) (Table 2). We identified the panel of SNPs significantly related to the diameter of each fiber type. Few polymorphisms affected fiber I and IIB diameters compared to those that were associated with percentage of the same fiber types (Table 3). Our results show that the most numerous polymorphisms were 3′UTR variants, while the least numerous variants in each histological trait (both parentage and diameter of all fiber types) were 5′UTR variants and missense mutations (Tables 2, 3). The list of all SNPs significantly associated with histological traits is presented in Supplementary Table S1 available online (goo.gl/VAHcUs).

Several polymorphisms/genes influenced more than one analyzed histological trait, simultaneously affecting the percentage and the diameter of the fibers. The identified panel of genes was the basis for selecting significant SNPs for subsequent GLM analysis performed on a larger population. From such genes, we selected the *TACC2*, *DEGS1*, and *FOXO1* genes, which were related to multiple histological properties.

### Genes mapping to QTLs

Based on the pig QTL database (pigQTLdb), DEGs (n = 355) identified by the RNA-seq, were located within QTLs related with fiber percentage and diameter (78 and 65, respectively). The most interesting locus seems to be on chromosome 14, at which 23 genes located in regions affecting percentage of I and IIA fibers were identified. DEGs localized in QTLs associated with percentage of fiber type IIB were identified mainly on chromosome 2 (SSC2) and SSC10 (Supplementary Table S2). The most represented QTLs related with muscle fiber diameter were identified on SSC12 and SSC15; SSC2, SSC14 and SSC15; SSC2 and SSC14 (diameter of fibers I, IIA, and IIB; respectively) (Supplementary Table S3). On chromosome 14, 13 DEGs associated with diameter of both fibers IIB and IIA were mapped to four QTL regions (Supplementary Table S3).

After the transcriptome-wide association study, 149 genes were selected (222 SNPs) and mapped to QTL regions. Results showed that chromosome 14, on which four QTLs were identified (related with percentage of fiber type I: QTLs #2822 and #2824; and IIB fiber type: #2823 and #7044), was the most overrepresented (Table 4). Also, on SSC10, four QTLs were identified, but these were represented by low numbers of genes (#7012 and #7026, percentage of fiber type I; #7031 and #7034, percentage of fiber type IIA) (Table 4). In the case of diameter of fiber, the higher numbers of genes were detected on SSC14 and SSC3 within QTL regions associated with diameter of fiber types IIA and IIB (Table 5).

### Association of selected SNPs with carcass characteristic and pork quality traits

Based on findings of other authors indicating relations of histological structure and meta quality and carcass traits [10–12], we also performed an association study on a large number of pigs. For this analysis four genes were selected: *TNNT2*; *FOXO1*; *TACC2*; *DEGS1*. These genes were previously identified as differentially expressed between muscle characterized by different histological properties; were localized within QTL regions associated with percentage of fiber types (*FOXO1*, IIB and IIA; *TNNT2* and *DEGS1*, I and IIA), and contained SNPs potentially related with muscle microstructure. Moreover, subsequent GLM analysis showed significant relationships of *FOXO1*, *DEGS1*, and *TNNT2* genes with loin ‘eye’ area, *FOXO1* with loin weight, as well as *FOXO1* and *TACC2* with lean meat percentage. Furthermore, the significant (p<0.01) effect of polymorphisms within *DEGS1*, *TACC2*, and *TNNT2* genes on IMF content was detected (Table 6). Only a small extent of SNPs was associated with pork color. *TACC2* polymorphism showed an effect on value of a* color parameter and *TNNT2* polymorphism influenced the b* color parameter (Table 6).

### Association of selected SNPs with histological microstructure of muscle tissue

The association study performed on a large number of animals (293 samples of *longissimus* muscle) confirmed significant effect of *FOXO1* and *TACC2* genes on histological characteristics in pigs. *FOXO1* was associated with diameter of fiber types IIA and IIB (p<0.01) (Table 7). The lowest diameter of both fiber types was detected in pigs with GG genotype, while the highest diameter was observed in heterozygote pigs. Conversely, a missense variant analyzed in the *TACC2* locus showed a significant effect on diameter of fibers I and IIA and percentage of fiber type IIB. Our results showed that pigs with the AA genotype were characterized by the lowest diameter of fibers I and IIA, as well as the lowest percentage of fiber type IIB (Table 7).

Furthermore, our result confirmed also significant correlation between selected histological traits and carcass characteristic. The diameters of all fiber types were positively, significant correlated with weight of loin. Diameters of fibers I and IIA was also correlated with lean meat percentage, while diameter of fibers I and IIB with meat color parameter, yellowness (b*) (Table 8). Percentage of fiber types was only corelated with loin ‘eye’ area (percentage of fiber I and IIB) (Table 8). For the other analyzed traits, the significant correlations were not obtained.

## DISCUSSION

Currently, much focus is placed on improving meat quality traits in pig breeding programs while maintaining satisfactory levels of meat and fatness characteristics [22]. Research has been performed on refining pork quality through different methods including environmental conditions (maintenance, nutrition), animal transport, slaughter procedures, and genetics. One of the factors determining pork quality is fiber types distribution in muscle tissue [23,24], which can be affected by genetic background [25,26]. However, the genetic basis of muscle fiber structure has not been well understood.

The present study is a continuation of previous research concerning identification of genes determining muscle fiber structure in pigs [13]. The differential expression analysis was performed on pigs representing one breed (Large White) to avoid an influence of breed factor on gene expression profile. The comparison of global gene expression profiles enabled us to detect the panel of genes that can play a role in formation of muscle fiber such as *TNNT2*, *FOXO1*, myosin XVIIIB, FERM domain containing 4B, and scavenger receptor class a member 5 [13]. The SNP calling analysis is a next step facilitating the search for candidate genes and establishing the exact genetic basis of phenotypic traits. In our research, the wide-transcriptome association study, performed as a preliminary analysis, allowed us to select the most important genetic variants potentially related with histological profile in muscle, the effects of which were validated on a large pig population.

Mapping both DEGs and SNPs significantly correlated with muscle microstructure to the known QTLs showed that a high number of genes were localized on SSC14. The most overrepresented were QTL regions associated with percentage of fiber type I (ID#2822 and #2824), IIB (ID#2823 and #7024), and diameter of fibers IIA (ID#7038) and IIB (ID#7018). These results were consistent with previous findings of Wimmers et al [26] (QTLs #2822; #2823; #2824) and Estelle et al [27] (QTLs #7018; #7024; #7038), who mapped QTLs associated with size and percentage of fibers on SSC14 and SSC15. These genome regions also span QTLs related with meat quality traits: pork color—redness and lightness [28], as well as fatness traits—abdominal fat weight and IMF content [29]. This may indicate that identified genes can affect some production traits in pigs.

Genome location was one of the selection criteria of genes for the subsequent GLM analysis. The association study carried out on a larger group of animals confirmed the significant effect of scrutinized polymorphisms in *FOXO1* and *TACC2* genes on some histological characteristics. A prior study [13] indicated differential expression of the *FOXO1* gene between pigs characterized by various percentages of fiber types I and IIB. The results obtained by transcriptome-wide association analysis, supplemented with information on genes localization in QTLs regions, were confirmed for both *FOXO1* and *TACC2*. The most interesting gene seems to be *TACC2* coding for transforming acidic coiled-coil protein 2. The RNA-seq approach enabled us to detect several polymorphisms within the *TACC2* gene. For the association study, the missense variant within *TACC2* locus was selected (p.Arg2613Cys) due to the highly potential results on protein structure, as well as biological function.

Our results show that investigated SNP in *TACC2* gene affected diameter of both fiber types I and IIA and percentage amount of fiber IIB. The same polymorphism was also significantly related with lean meat percentage as well as IMF content. In the cell cycle, *TACC2* protein is involved in G2/M progression [30] by playing a key role in microtubule dynamics. It has been established that the *TACC2* gene is a critical factor controlling mitotic spindle dynamics during the normal course of the mitosis process [30]. To date, scarce research has investigated the involvement of the *TACC2* gene in fiber type specification or muscle growth, even though this gene is highly expressed in skeletal and cardiac muscle. Our study suggested that transforming acidic coiled-coil protein 2 could play an important role during muscle fiber differentiation, resulting in modification of the number and size of muscle fibers. In turn, the differences in histological features are related to muscle mass and/or meat quality traits. The fiber composition in muscle is related with fatty acid and glycogen content due to the type of energy sources. The fiber type I is characterized by high fatty acid storage and low glycogen content, whereas the opposite trend is observed for the fiber type IIB. The observed missense variant changing arginine to cysteine in two different *TACC2* isoforms (ENSSSCP00000029 474.1:p.Arg795Cys; ENSSSCP00000025150.1:p.Arg2613Cys) may affect function of synthesized proteins. Our results confirmed previous findings that percentage amount of type fiber IIB is inversely related with IMF [11]. According to the *TACC2* missense variant, pigs with the AA genotype had significantly lower numbers of fiber type IIB and higher IMF content.

Conversely, our results indicated significant association between a *FOXO1* mutation and diameter of fibers IIA and IIB and carcass characteristics such as weight of loin, loin ‘eye’ area and lean meat percentage. Furthermore, the significant correlation was observed between diameter of all fiber types and weight of loin. A previous study [13] showed differential expression of the *FOXO1* gene depends on histological microstructure of muscle tissue. Moreover, our current research, which confirmed a significant relationship of a 3′UTR variant in the *FOXO1* gene with fiber diameter, suggests that the analyzed SNP can affect *FOXO1* transcript levels and histological profile as a result. Schiaffino and Mammucari [31] showed that FOXO proteins can regulate skeletal muscle growth via interaction with the IGF1-Akt/PKB pathway. Moreover, the research performed on transgenic mice confirmed that overexpression of the *FOXO1* gene leads to muscle atrophy and increases transcript level of cathepsin L [32]. Researchers demonstrated that skeletal muscle of mice overexpressing *FOXO1* was characterized by decrease size of both fiber types I and II, likewise reducing the number of fiber type I. Subsequently, the up-regulation of the cathepsin L gene can indicate increasing protein degradation processes in muscle tissues. The *FOXO1* gene has been considered as a main genetic factor closely associated with muscle fiber type specification and skeletal muscle differentiation [33]. Our results confirmed that in pigs, the *FOXO1* gene is related to muscle mass, as well as size of fiber types IIA and IIB, and should be investigated in the future for relation with important production traits.

Our results did not show the significant association of *DEGS1* and *TNNT2* genes with histological traits. However, polymorphisms identified in both genes affected loin ‘eye’ area and lean meat content in carcasses. This suggests that detected SNPs can be related with muscle growth processes but do not affect histological structure of muscle tissue at the same time. Furthermore, there is also the possibility of occurrence of another polymorphism within the *DEGS1* and *TNNT2* loci (in promoter or intron regions), which are associated with gene expression, and as a result, with cell size and fiber type distribution.

## CONCLUSION

The use of RNA-seq data enabled us to identify a panel of SNPs within coding and UTRs regions with potential effects on histological microstructure. The genes detected by differential expression and the transcriptome-wide association analyses enabled us to select candidate genes potentially related to distribution of fiber types in porcine muscle tissue. These results can inform future studies evaluating the use of selected SNPs as genetic markers related to muscle histological profile and production traits in pig breeding.

## Supplementary Data



## Figures and Tables

**Table 1:** Detailed information about the analyzed genes and SNPs along with the methods used

Gene	Accession number	SNP	SNP variant	Primers	Endonuclease	Obtained alleles (bp)
*TNNT2*	ENSSSCG00000023031	c.294T>C (rs330309370)	splice region variant/synonymous variant	F CACGCCTCCTTCTGTTCATC R CAGCTCCTCCTCCTCCTTCT	*HpyCH4IV*	T: 334 C: 255, 79
*FOXO1*	ENSSSCG00000009370	c.*1395A>G (rs337841578)	3′ prime UTR variant	F TCAGCAAGTTTTCCCTCTGTaCT R CCCCAGCTTAACTTTCCAGA	*HpyCH4III*	A: 154 G: 131, 23
*TACC2*	ENSSSCG00000028063	c.7837C>T p.Arg2613Cys (rs80840610)	missense variant	F GCTGAGGTGTTCGTTGTGAA R TGCACGATTGACGCTTTTAG	*AluI*	C: 200, 121, 56, 16 T: 200, 89, 56, 32, 16
*DEGS1*	ENSSSCG00000024484	c.*250T>C (rs344988843)	3′ prime UTR variant	F AGACGAGCCCCTCGTACAAT R GAACTCCCAGGCCTTTTCA	*HpyCH4IV*	T: 361 C: 211, 150

SNP, single nucleotide polymorphism; *TNNT2*, troponin T2; *FOXO1*, forkhead box O1; *TACC2*, transforming acidic coiled-coil-containing protein 2; *DEGS1*, delta 4-desaturase, sphingolipid 1.

**Table 2:** Number of identified polymorphisms and selected genes significantly associated with percentage of all fiber types

Fiber type	SNPs	Gene (N)	Genetic variants	SNPs (N)	Genes
I	560	630	5 prime UTR variant	19	*MAFF; SCO2; MRTO4; SDHB; CD151; MRTO4*
			Synonymous variant	150	*MYH7; COL5A1; SETX; TNNI1; ACE; MYH3; SLC15A4; SLC25A37; ATIC; CAST; CNOT11;CHD7*
			Missense variant	66	*SLC16A5; MYO18B; FBXO7; DECR1; PINK1; SLC16A5; HEBP2*
			3 prime UTR variant	300	*SLC17A5; MYO1E; TGFB2; DEGS1; SLC16A6; RPTOR; TACO1; BMP1; BMP6; MTR; TMX2; ILF2; GLO1*
			Non coding transcript exon variant, non coding transcript variant	25	*TXLNA; SSH2; MYH7; GPX3; RPIA; ACE; SGCA*
IIA	251	299	5 prime UTR variant	10	*TMED7; UQCC3; GTF2F1*
			Synonymous variant	80	*MYBPC1; PER1; ITGB7; BIN1; TRRAP; ITGB7; LIN7B; SUN2*
			Missense variant	31	*TACC2; PROB1; MLH1; HCR; PISD; SERPINB1*
			3 prime UTR variant	127	*CASP2; BMPER; MAFB; VIPR1; SLC9A3R1; COL4A2; PPP1R9B; CRP*
			Non coding transcript exon variant, non coding transcript variant	3	*TACC2; ZCCHC6*
IIB	837	921	5 prime UTR variant	33	*SLC2A12; MARC2; AQP3; ACO1; MYH13; GOT1; TACC2; MYLK2;*
			Synonymous variant	261	*TACC2; APP; PLCD1; MYH13; TSN; GOT1; MYLK2; BRAF; SLC43A2; COL4A1; MARC2*
			Missense variant	91	*TACC2; MARCH6; LTF; MTO1; CAST; FYCO1; LIFR*
			3 prime UTR variant	439	*DEGS1; FOXO1; AKT3; FYN; RPTOR; SCARA5; BMPR2; SRFBP1; TAGLN2; TGFB1; GLO1*
			Non coding transcript exon variant, non coding transcript variant	13	*TACC2; SSH2; LAT; ARNT; KLRB1; TNFRSF21*

N, number of identified genes; SNP, single nucleotide polymorphisms.

**Table 3:** Number of identified polymorphisms and selected genes significantly associated with diameter of all fiber types

Fiber type	SNPs	Gene (N)	Genetic variants	SNPs (N)	Fiber type
I	91	112	5 prime UTR variant	6	*MYL9; TMEM258; MAP2K2*
			Synonymous variant	29	*TACC2; GPX3; CA3; HP; NOB1; MYH7; PIK3C3; ATAT1*
			Missense variant	14	*TACC2; FBXO7; BCAR1; HSF4; ELL2; TMEM5*
			3 prime UTR variant	38	*ESR1; ERG; ITGB1; TACC2; MARCH6; TTL; CHD1L; HP; HYI; ATAT1*
			Non coding transcript exon variant, non coding transcript variant	4	*TACC2; SGCA*
IIA	139	162	5 prime UTR variant	5	*CAPN3; PFDN4; MAFF; NOL10; FGFR1OP2; INTS3*
			Synonymous variant	42	*ACE; TACC2; ERBB2; NRBF2; HPS6; E2F6; SUN2; ILF2; RYR1; ENO1; AARS;*
			Missense variant	15	*TACC2; SIRT3; ACAT2; FLAD1; RFX5; C4A; GSTM3*
			3 prime UTR variant	72	*FOXO1; ITGB1; STX7; OAS2; CD82; HOXC6; PRR3; SLC29A1; STK38; RPTOR;*
			Non coding transcript exon variant, non coding transcript variant	5	*TACC2; ACE; SGCA; CD8A*
IIB	33	39	5 prime UTR variant	1	*TMEM258*
			Synonymous variant	15	*TRRAP; HP; HK1; SLC35B1; NXN; ADAM17; LUM*
			Missense variant	3	*TACC2; BCAR1; HEATR5A*
			3 prime UTR variant	14	*GOT1; OTULIN; GPAM; YBX3; SMUG1; IDH3A; ZSCAN31*

N, number of identified genes; SNPs, single nucleotide polymorphisms.

**Table 4:** Genes obtained after transcriptome-wide association study mapped to QTL regions related to percentage of fiber types I, IIA, and IIB

I fiber type	SSC	QTL ID			IIA fiber type	SSC	QTL ID		IIB fiber type	SSC	QTL ID		
		
*RGP1*	1	2794			*CCDC28A*	1	7015		*VPS36*	11	7020	7023	7042
*IKBKAP*		2794			*KCNQ5*		7015		*WRAP53*	12	7021	7036	
*MARC2*	10	7012	7026		*APIP*	2	4025		*DHRS7C*		7021	7036	
*C1orf115*		7012	7026		*RPS13*		4025	7016	*ANAPC7*	14	2823	7024	
*DEGS1*		7012	7026		*DHPS*		4025	7016	*PPTC7*		2823	7024	
*RABIF*		7012	7026		*MARC2*	10	7034		*AP1B1*		2823	7024	
*C9orf64*		7012	7026		*C1orf115*		7034		*MTR*		2823	7024	
*ANAPC7*	14	2822	2824		*DEGS1*		7034		*ARV1*		2823	7024	
*PPTC7*		2822	2824		*RABIF*		7034		*RAB4A*		2823	7024	
*AP1B1*		2822	2824		*C9orf64*		7031		*SUPV3L1*		2823	7024	4045
*MTR*		2822	2824		*NRP1*		7031		*EXOSC1*		2823		
*ARV1*		2822	2824		*VPS36*	11	7030		*GOT1*		2823		
*RAB4A*		2822	2824		*MAP3K14*	12	7033		*FAM45A*		7044		
*SUPV3L1*		2822	2824	4022	*TUBG2*		7033		*TACC2*		7044		
*EXOSC1*		2822	2824		*NKIRAS2*		7033						
*GOT1*		2822	2824		*ZNF385C*		7033						
					*CNP*		7033						
					*TTC25*		7033						

QTL, quantitative trait loci. SSC, chromosome 2.

**Table 5:** Genes obtained after transcriptome-wide association study mapped to QTL regions related to percentage of fiber types I, IIA, and IIB

I fiber type	SSC	QTL ID		IIA fiber type	SSC	QTL ID	IIB fiber type	SSC	QTL ID	
		
*SCRN2*	12	2819	2821	*SLF1*	2	2797	*RGP1*	1	2795	
*UTP18*		2819	2821	*CAST*		2797	*IKBKAP*		2795	
*TOM1L1*		2819	2821	*ANAPC7*	14	7018	*TTL*	3	7037	
*WRAP53*		2819	2821	*PPTC7*		7018	*UXS1*		7037	
*FASTKD1*	15	2832		*AP1B1*		7018	*IL1R1*		7037	
*HNRNPA3*		2832		*MTR*		7018	*MAP4K4*		7037	
				*ARV1*		7018	*NAGK*		7037	
				*RAB4A*		7018	*PCYOX1*		7037	
				*SUPV3L1*		7018	*EFEMP1*		7037	
				*FAM45A*		2827	*MTIF2*		7037	
				*TACC2*		2827	*FEZ2*		7037	
				*PAF1*		2827	*CRIM1*		7037	
							*HDGF*	4	2807	2810
							*PAQR6*		2807	2810
							*RFX5*		2807	2810
							*ANAPC7*	14	7038	
							*PPTC7*		7038	
							*AP1B1*		7038	
							*MTR*		7038	
							*ARV1*		7038	
							*RAB4A*		7038	
							*SUPV3L1*		7038	
							*FAM45A*		2828	
							*TACC2*		2828	
							*PAF1*		2828	

QTL, quantitative trait loci; SSC, chromosome 2.

**Table 6:** Association of analyzed polymorphisms with carcass and meat quality traits

Items	*FOXO1*		*DEGS1*		*TACC2*		*TNNT2*	
			
	Genotype	LSM±SE	Genotype	LSM±SE	Genotype	LSM±SE	Genotype	LSM±SE
Weight of loin (kg)	AA	6.07±0.03^a^	TT	6.11±0.04	CC	6.06±0.08	TT	6.07±0.02
	AG	6.11±0.04^a^	CT	6.03±0.03	CT	6.11±0.03	TC	6.11±0.07
	GG	5.93±0.10^b^	CC	6.05±0.03	TT	6.02±0.03	CC	5.96±0.27
Loin ‘eye’ area (cm)	AA	52.6±0.26^b^	TT	53.3±0.52^a^	CC	52.8±0.84	TT	53.3±0.25^ab^
	AG	54.7±0.54^a^	CT	53.7±0.46^a^	CT	54.0±0.42	TC	54.9±0.87^a^
	GG	53.5±0.12^ab^	CC	52.1±0.33^b^	TT	52.8±0.32	CC	51.7±3.10^b^
Lena meat percentage	AA	61.4±0.14^b^	TT	61.5±0.27	CC	60.6±0.38b	TT	61.4±0.13
	AG	61.5±0.23^b^	CT	61.8±0.18	CT	61.5±0.19^ab^	TC	62.8±0.35
	GG	62.7±0.04^a^	CC	61.4±0.20	TT	61.9±0.17^a^	CC	61.5±0.78
IMF	AA	1.40±0.02	TT	1.36±0.06^B^	CC	1.44±0.09^A^	TT	1.35±0.02^C^
	AG	1.36±0.03	CT	1.37±0.03^B^	CT	1.39±0.03^AB^	TC	1.56±0.08^B^
	GG	1.38±0.11	CC	1.46±0.03^A^	TT	1.35±0.03^B^	CC	2.00±0.31^A^
L*	AA	54.15±0.12	TT	54.10±0.22	CC	53.64±0.51	TT	54.2±0.11
	AG	54.03±0.21	CT	54.06±0.14	CT	54.06±0.16	TC	53.4±0.30
	GG	54.01±0.45	CC	54.30±0.20	TT	54.25±0.13	CC	52.7±0.96
a*	AA	16.89±0.08	TT	16.84±0.14	CC	17.21±0.06^a^	TT	17.03±0.08
	AG	17.02±0.14	CT	17.00±0.10	CT	17.07±0.12^ab^	TC	16.91±0.20
	GG	17.04±0.38	CC	16.84±0.14	TT	16.80±0.09^b^	CC	17.04±0.73
b*	AA	3.24±0.10	TT	3.38±0.17	CC	3.82±0.40	TT	3.46±0.10^b^
	AG	3.41±0.18	CT	3.25±0.12	CT	3.42±0.14	TC	3.23±0.19^b^
	GG	3.58±0.54	CC	3.26±0.19	TT	3.14±0.12	CC	5.23±1.37^a^

*FOXO1*, forkhead box O1; *DEGS1*, delta 4-desaturase, sphingolipid 1; *TACC2*, transforming acidic coiled-coil-containing protein 2; *TNNT2*, troponin T2; LSM, least squares mean; SE, standard error; IMF, intramuscular fat content; a*, b*, L*, meat colour parameters.

a,b p<0.05;

A,B,C p<0.01.

**Table 7:** Association of selected polymorphisms with muscle microstructure traits

Items	*FOXO1*		*DEGS1*		*TACC2*		*TNNT2*	
			
	Genotype	LSM±SE	Genotype	LSM±SE	Genotype	LSM±SE	Genotype	LSM±SE
Percentage of fiber type								
I	AA	12.3±0.33	TT	12.6±0.44	CC	12.4±0.90	TT	12.4±0.26
	AG	12.3±0.42	CT	12.5±0.37	CT	12.1±0.34	TC	12.6±0.98
	GG	12.2±1.11	CC	12.0±0.50	TT	12.7±0.39	CC	11.1±2.62
IIA	AA	16.8±0.45	TT	17.1±0.53	CC	15.9±0.97	TT	16.3±0.33
	AG	16.4±0.47	CT	16.7±0.54	CT	15.7±0.41	TC	16.6±0.86
	GG	15.8±0.84	CC	15.8±0.45	TT	17.4±0.50	CC	12.4±4.04
IIB	AA	71.3±0.48	TT	70.3±0.71	CC	67.5±1.60^b^	TT	71.2±0.38
	AG	71.3±0.65	CT	71.2±0.54	CT	73.2±0.55^a^	TC	70.7±1.34
	GG	71.9±1.44	CC	72.1±0.69	TT	71.6±0.51^a^	CC	76.4±6.60
Diameter of fiber type								
I	AA	54.4±0.69	TT	55.2±1.10	CC	52.0±1.35^b^	TT	55.3±0.55
	AG	56.2±0.84	CT	55.4±0.81	CT	55.4±0.88^a^	TC	55.2±1.64
	GG	53.3±2.50	CC	55.1±0.89	TT	55.8±0.76^a^	CC	56.9±8.01
IIA	AA	55.8±0.63^A^	TT	55.8±1.00	CC	54.0±1.73^B^	TT	56.0±0.50
	AG	56.9±0.83^A^	CT	55.5±0.73	CT	56.2±0.79^A^	TC	54.3±1.54
	GG	50.6±1.78^B^	CC	56.3±0.78	TT	55.7±0.64^AB^	CC	55.9±5.06
IIB	AA	71.6±0.82^AB^	TT	71.3±1.24	CC	67.5±1.80	TT	71.9±0.66
	AG	73.6±1.07^A^	CT	72.2±0.97	CT	73.2±1.01	TC	71.6±1.97
	GG	67.0±2.44^B^	CC	71.3±1.03	TT	71.6±0.88	CC	75.4±5.60

*FOXO1*, forkhead box O1; *DEGS1*, delta 4-desaturase, sphingolipid 1; *TACC2*, transforming acidic coiled-coil-containing protein 2; *TNNT2*, troponin T2; LSM, least squares mean; SE, standard error.

a,b p<0.05;

A,B p<0.01.

**Table 8:** The obtained significant correlation coefficients between percentage or diameter of fiber types and carcass characteristics

Items	Weight of loin (kg)	Loin eye area (cm)	Lean meat percentage	b*
Percentage of fiber type				
I	ns	−0.20**	ns	ns
IIA	ns	ns	ns	ns
IIB	ns	0.12*	ns	ns
Diameter of fiber type				
I	0.12*	ns	0.15*	0.20**
IIA	0.21**	ns	−0.17*	ns
IIB	0.15**	0.11*	ns	0.16*

b*, yellowness; ns, not significant.

* p<0.05;

** p<0.01.
