# Supplementary material for: Screening for candidate genes related with histological microstructure, meat quality and carcass characteristic in pig based on RNA-seq data
Source: Asian-Australas J Anim Sci. 2018 Mar 13;31(10):1565–74. doi: 10.5713/ajas.17.0714 (PMC6127584; doi:10.5713/ajas.17.0714)
Supplement: Supplementary file 1 [file ajas-31-10-1565-supplementary.pdf]

Table S1. Selected polymoprphisms and corresponding pvalue associated with histological properties.

SSC	SNP id	position	Histological traits					
			percentage of I fiber type	percentage of IIA fiber type	percentage of IIB fiber type	diameter of I fiber type	diameter of IIA fiber type	diameter of IIB fiber type
1	rs3409994	305457149	0.00335248042790876	0.0590634885573476	0.00152043845442082	0.177017398245816	0.183401953136455	0.274134039995061
1	rs3413903	59275519	0.00534371626956334	0.0328294408373238	0.00107310527351659	0.112200317611478	0.142801551192937	0.269173639487387
1	rs3218579	14450509	0.00556124696577777	0.0716640573508268	0.00139762500842817	0.0163113812279531	0.0857576546130285	0.137598770338538
1	rs3450452	264195414	0.00590704228054546	0.122736337656803	0.00269524044107494	0.0595594716440999	0.132092875050723	0.215781647990169
1	rs3341760	91996316	0.00670337160209689	0.337663754061732	0.0116286597011556	0.173589541989924	0.0992024294801522	0.293074854373553
1	rs3243255	306730317	0.00790083948040698	0.190845910716568	0.0107928886355426	0.18008675817127	0.085014182310868	0.311662484472031
1	rs8135113	28796261	0.00900817676074971	0.215527310303848	0.0113503377253333	0.191655146996718	0.0921008401389852	0.108837474871834
1	rs8134861	16463222	0.0123690513707778	0.0454347723867294	0.00986875514502993	0.30408358341069	0.0592205829132902	0.140336549751768
1	rs1072088	82059840	0.0136373481565458	0.18687846063932	0.00808365505732643	0.0986072808403489	0.263900407409718	0.473442636802575
1	rs3400291	180763523	0.0139686660007038	0.154075746765721	0.0104678955220412	0.147652374609822	0.0702952249741271	0.281726641026217
1	rs3403438	200362379	0.0139686660007038	0.154075746765721	0.0104678955220412	0.147652374609822	0.0702952249741269	0.281726641026217
1	rs3338776	146065178	0.0139686660007038	0.154075746765722	0.0104678955220412	0.147652374609823	0.0702952249741266	0.281726641026216
1	rs6972688	3236305	0.0141186362400733	0.285604962094647	0.000549229732830458	0.230814352484578	0.1543302517026	0.41172608602669
1	rs7139829	283266342	0.014437348492521	0.180851913226087	0.00682966615989721	0.11055400810467	0.172023172204587	0.224124035675177
1	rs3231310	215796655	0.0153118711904875	0.235654131160917	0.00727366690542296	0.203980306654125	0.911772500828247	0.384673601416743
1	rs3331455	87145893	0.0163966731579355	0.0663658416352514	0.00269341649348695	0.0440998424838027	0.061241805338725	0.12457786507978
1	rs3218657	126385263	0.0171387467852789	0.208716703553595	0.00512186716322155	0.103773536020519	0.0997602690099982	0.229193940260208
1	rs8086827	201974454	0.0227843188613178	0.160941933205903	0.00701489333611939	0.302775027330461	0.36494371327024	0.706378819763557
1	rs3398274	86046817	0.0234425696891336	0.281783036966105	0.00878042397437894	0.00689351779760885	0.0539603952318186	0.153313709093762
1	rs1072088	82059783	0.023544647094557	0.0287404758798913	0.000540984266270709	0.356153148478527	0.531498917636717	0.713093087970178
1	rs3189881	142983928	0.023603626223908	0.0513125921509423	0.00936952617433432	0.0452756769836591	0.0743213628565946	0.0834607284387265
1	rs8097402	64129801	0.0242176093148346	0.126573728595235	0.0106152699635386	0.110447517769429	0.0566863687318889	0.278534406804127
1	rs8135231	294017568	0.0242239609799528	0.0947911219857082	0.00892462528786562	0.15574621642309	0.102829850735413	0.358968696143399
1	rs3185702	306985788	0.0263425218560985	0.192187132755502	0.0113780484165758	0.196974050636615	0.138806385967855	0.204247459205859
1	rs3451284	267559187	0.027775700633076	0.124844269428571	0.0080949583319271	0.101937464120971	0.154801303909541	0.228780567722854
1	rs3436942	304422050	0.0295902906644405	0.1082009641437	0.00357230990764319	0.118357461495387	0.0670267087556335	0.157134067439611
1	rs3274379	86038450	0.0296363117896679	0.029310657436627	0.00913846492832062	0.120020838288653	0.190511143675014	0.279701054417822
1	rs3442755	144958551	0.0304306528187477	0.307747071661373	0.00576424265962728	0.150206986783962	0.128449661622279	0.314097440089236
1	rs3456667	84368788	0.0305511572115682	0.242730351449853	0.00616479787691409	0.102475187255201	0.126630418070983	0.309569531611924
1	rs3256811	305270382	0.0316940248989867	0.0653223920694986	0.00834048867970987	0.0857496453212151	0.213182783763782	0.24921941635342
1	rs3433903	37327570	0.0325681726215984	0.324074111970198	0.00401317868352987	0.383143460227735	0.426380778618272	0.629281520203006
1	rs3211598	283051952	0.0336207539793486	0.0538441291662035	0.00207430673889234	0.115239688699538	0.0863809885715403	0.417579331816943
1	rs3255664	141721981	0.0345298456671876	0.192987690343411	0.0118011627940432	0.0547553095693234	0.0521821717632775	0.0904884140453482
1	rs3317176	143930360	0.0350647768171321	0.0498994487401938	0.00713825151606574	0.150593934833108	0.253947888666732	0.356098301047794
1	rs3456746	248827966	0.0358917632856172	0.0463345238391749	0.00639889060731786	0.128233364614229	0.17958598853555	0.281475102265858
1	rs3256284	50175777	0.0375779243975934	0.0351822338824981	0.00340490323860668	0.161205111071998	0.20944981890252	0.360492213288616
1	rs3260460	307476242	0.0395656093097751	0.283824348684009	0.00976273767078435	0.136276086642544	0.171135384305977	0.32283546035247
1	rs3252205	10907361	0.0401804745345199	0.0458895407665772	0.00510057381261988	0.128075760966858	0.190905623053819	0.280324635910453
1	rs3453876	18591467	0.0411509968297415	0.0874495990068728	0.0076116574198672	0.136200864962756	0.135606973745208	0.276971262970254
1	rs3231002	83410803	0.0420367454474066	0.0569350072407941	0.00134853161814629	0.0737279887192004	0.210359129238667	0.343423792435016
1	rs3239221	208238129	0.0423439049166626	0.0305292216582322	0.0051972576494507	0.100547004233657	0.158159406943658	0.241950145925271
1	rs3415133	74422533	0.0423439049166626	0.0305292216582322	0.0051972576494507	0.100547004233657	0.158159406943658	0.241950145925271
1	rs3324891	132678722	0.0458445550070332	0.444300498692909	0.0064276691844358	0.202546642720584	0.165108000897881	0.241749135731994

1	rs3229582	126384135	0.050195196130505	0.364985919700094	0.0097606077252325	0.192612164191449	0.0707595221855232	0.317887858228313
1	rs3371340	33358974	0.0525360153682245	0.0477224018521101	0.00707061102703598	0.126310137901548	0.197482880148354	0.268897621720468
1	rs3343673	284184921	0.0525481495687543	0.036870868386024	0.00648164564267231	0.0339360476967438	0.136947444722901	0.132290705859013
1	rs3318624	311559345	0.0532857838341765	0.187071772804572	0.00608690476651739	0.207159200538886	0.11510622956422	0.1825857043477
1	rs3228934	298943465	0.0570911059344998	0.0733127242002763	0.00074259455806941	0.324092046223651	0.283899613410231	0.389410144651597
1	rs3233694	50175211	0.0588179533518426	0.00869512614128417	0.00943752566980732	0.111887056861693	0.189169393640826	0.281019139187999
1	rs3221211	280510302	0.0596706186628109	0.0595285903192677	0.00370041132753074	0.0904152907544914	0.00642954864873064	0.0658286505881013
1	rs8087483	283609728	0.0597208563833915	0.0733059398999409	0.00604941757489226	0.122378501925278	0.125567246952733	0.437324548804881
1	rs8089874	283610145	0.0597208563833915	0.0733059398999409	0.00604941757489226	0.122378501925278	0.125567246952733	0.437324548804881
1	rs3386789	103504169	0.0668386469747465	0.0757715303270151	0.00708367881138721	0.132400086443693	0.0585641748203113	0.48590236069646
1	rs3269321	302299142	0.0774744747787648	0.0377397121946279	0.0048814035371879	0.15114604014676	0.226149445379375	0.251174784182435
1	rs3332553	313878617	0.0785213558143476	0.0579412657042113	0.0106625309826887	0.162670897316064	0.256735177556632	0.316891008464154
1	rs3249652	271655971	0.0785213558143476	0.0579412657042113	0.0106625309826888	0.162670897316064	0.256735177556632	0.316891008464154
1	rs3251776	303946538	0.0833572426286648	0.0646261554176018	0.00833087608991776	0.106619574340334	0.105707163296285	0.31396721198138
1	rs3409745	118997548	0.0840728539617639	0.419045590384662	0.0105354748516387	0.330275113979485	0.039215397572251	0.490275852005972
1	rs7938770	157879748	0.0859243436465148	0.0286945674215644	0.00716162588888384	0.0397826208708378	0.0488587523799102	0.180391741995843
1	rs3190361	245582084	0.0859698013950741	0.219044170588514	0.00930378561843529	0.0376423463673231	0.106838362434827	0.203516709541196
1	rs7933040	91872944	0.0979735073202697	0.12606293028056	0.00295813504173352	0.231777748493965	0.0458747866637897	0.128166146665249
1	rs8083207	252997864	0.124196799809035	0.51868744117301	0.0107596382028973	0.103261581762071	0.278524984680196	0.243424619930044
1	rs3289319	123368603	0.151616239895891	0.20057407206482	0.00684861868159911	0.642690128127468	0.489760331727337	0.413207014740279
1	rs3230006	39462786	0.161663273915889	0.248802248744993	0.000659022237053444	0.41216016070897	0.0432419954153656	0.127242580625542
1	rs7033636	14442593	0.16996831549622	0.0675621415595887	0.00468831389526478	0.286677915199037	0.214961795436148	0.537068711174211
1	rs6904529	292357382	0.215804987656697	0.358337991976188	0.00827010104802004	0.456170869147153	0.283882973843368	0.303582616171078
1	rs8135299	305503624	0.720496761339335	0.653976865607154	0.0107697445655904	0.395296551122662	0.306102995848485	0.0643157350936329
2	rs3241619	10488771	0.000462648219101227	0.199455384163147	0.00449563664759079	0.324844423401514	0.467212947974308	0.551780447178
2	rs3340601	78832617	0.00119700607701089	0.117647303855513	0.00143132146578057	0.228127948082776	0.291502194412751	0.374676949083733
2	rs3189424	13408594	0.00120645409859502	0.227526891524948	0.0113671813104878	0.0631558275119207	0.0671548217606542	0.1372782438211
2	rs7090508	149740319	0.00128174968787085	0.0211177868630719	0.000499751485240913	0.0969906042298176	0.0912879183133848	0.231476502114981
2	rs3390362	104919154	0.00207368479333776	0.0122338518728085	0.00156445174978068	0.367980032803344	0.361966233161177	0.434475401853545
2	rs3457654	159561482	0.00207368479333777	0.0122338518728086	0.00156445174978071	0.367980032803344	0.361966233161178	0.434475401853544
2	rs3251740	107114719	0.00390142684199296	0.164258758021778	0.00209357311758126	0.0147904745189411	0.137682494779063	0.0542337065413342
2	rs3429505	107363650	0.00390142684199296	0.164258758021778	0.00209357311758126	0.0147904745189411	0.137682494779063	0.0542337065413342
2	rs1969497	120042893	0.00463725341781436	0.165240419096315	0.00762708421314868	0.0890074204669223	0.0422930600404913	0.149395452729857
2	rs3338220	149015	0.00493183298638927	0.0130518521873758	0.000266701206576119	0.112156701566722	0.105374011624018	0.218902298256173
2	rs3351743	127982591	0.00549908588795064	0.0223136108469875	0.0038806278263861	0.141801227127494	0.213224941993757	0.292032873199954
2	rs8121843	28481463	0.00726369716801976	0.0533117240319091	0.000316020169650435	0.135115261816779	0.104570561208607	0.268966200316102
2	rs3350716	145962340	0.00736070926762321	0.220954238655141	0.00357298928534615	0.151548240062725	0.129974733679853	0.276740237019097
2	rs3217353	149745241	0.00761990357185629	0.249877150151785	0.0109131446453263	0.0583995913694207	0.0107227859817902	0.0634927563101771
2	rs7029582	66682742	0.00935322307459372	0.136129762522743	0.00567153823162922	0.330313856536424	0.242904846274216	0.218051678538774
2	rs3364764	28482170	0.00992727580016529	0.0385927531949261	0.00109233751586767	0.171158782785315	0.119132933766505	0.317012444647259
2	rs3237031	12882190	0.0107683503441062	0.143420738642906	0.00370861356408462	0.0842037575574427	0.0294463829822967	0.226193239832709
2	rs3274591	12882978	0.0107683503441062	0.143420738642906	0.00370861356408462	0.0842037575574427	0.0294463829822967	0.226193239832709
2	rs3351036	12882908	0.0107683503441062	0.143420738642906	0.00370861356408462	0.0842037575574427	0.0294463829822967	0.226193239832709
2	rs3280597	61021987	0.0123203623003067	0.154658063582556	0.00293086295240171	0.184415471768388	0.088081445348883	0.323405708109651
2	rs3415947	6275375	0.013484757094475	0.397417146754799	0.00462710657408809	0.296177562031267	0.159191167018881	0.393665677977919
2	rs3251804	138701382	0.0139686660007038	0.154075746765721	0.0104678955220412	0.147652374609822	0.0702952249741269	0.281726641026217

2	rs3352991	4408188	0.0143288807269363	0.0932682163431512	0.000305126909839114	0.205593216353807	0.157131701537014	0.345149642320848
2	rs3209660	71775465	0.0143288807269364	0.0932682163431511	0.000305126909839114	0.205593216353807	0.157131701537014	0.345149642320847
2	rs3328671	153885049	0.0144179004913015	0.0952823563640165	0.00970676118800461	0.307172065295611	0.172651357910909	0.23850245773004
2	rs6999781	16548168	0.0164250602488031	0.183363185640186	0.00542203777196014	0.0831940186550725	0.0312256923561628	0.110893873746819
2	rs3345586	52014704	0.0171621282917609	0.0682461588525993	0.00825343521196662	0.209146299289763	0.0993760987224283	0.334851551086571
2	rs3391921	10168053	0.0179710510859965	0.287311289628177	0.0101785391933175	0.14083754759941	0.144152234150619	0.371022331868033
2	rs3432070	157282686	0.0181275653568287	0.15330033443133	0.0108620762536782	0.0765935172937554	0.0626451835742366	0.11619655915549
2	rs3388240	65401456	0.0182415018496304	0.182854577314135	0.000778787749655973	0.230765025792444	0.311667841695452	0.439422513582524
2	rs3217509	12131461	0.0207147127269841	0.398978242852886	0.00392204791223662	0.0897934544956558	0.107375553200132	0.251862462317142
2	rs7015205	52047674	0.0217934268583884	0.0389858509572832	0.00126134160680166	0.0670018728535395	0.0999641599680247	0.260145379753215
2	rs6938676	6953607	0.021824969003032	0.0621528830068201	0.00160652676816406	0.294120831231042	0.219153466125978	0.574270088055224
2	rs5561828	147318843	0.0218282397265991	0.0584785877254089	0.00269169452604559	0.121470589245747	0.151749555570811	0.312120628515062
2	rs3439390	149746681	0.0218419699488899	0.0233927761628423	0.00157946256163068	0.0142912698749525	0.0496171442393462	0.139775188004665
2	rs3386414	9241201	0.0226516027076687	0.327161021128783	0.0321811177757073	0.00193347240209044	0.0250535311795251	0.00515697209139389
2	rs3387175	9192543	0.0226516027076687	0.327161021128783	0.0321811177757073	0.00193347240209044	0.0250535311795251	0.00515697209139389
2	rs3447398	9186577	0.0226516027076687	0.327161021128783	0.0321811177757073	0.00193347240209044	0.0250535311795251	0.00515697209139389
2	rs3443972	148327971	0.0234073476589881	0.210599061443396	0.0114838435421427	0.0133768875083331	0.042507229235867	0.0328513719429441
2	rs6917516	8328525	0.0262496250638064	0.0152879585799422	0.00518632132210136	0.0817029100852417	0.174657862939988	0.199870049127405
2	rs3410488	142608506	0.0281369354327813	0.0334822001252552	0.00273459287178072	0.0800759446173637	0.148143367879294	0.207424198517441
2	rs3217195	124611830	0.0289313219262311	0.130962159927636	0.00127520298749568	0.159505215414417	0.021322271230145	0.197522391856187
2	rs6985894	29200173	0.0289313219262311	0.130962159927636	0.00127520298749569	0.159505215414417	0.021322271230145	0.197522391856188
2	rs3431655	10465972	0.0294853919371986	0.0951640149960953	0.00684799268168592	0.0230384413702044	0.0534956523733169	0.073291756860693
2	rs3396116	142687520	0.0305511572115682	0.242730351449853	0.00616479787691406	0.102475187255201	0.126630418070983	0.309569531611924
2	rs7109082	142695696	0.0305511572115682	0.242730351449853	0.00616479787691406	0.102475187255201	0.126630418070983	0.309569531611924
2	rs3189581	78770722	0.0314625092077963	0.131275741085077	0.00106351832324044	0.159083420473214	0.0343811937831793	0.218371764010871
2	rs3268539	77487748	0.0326180406184519	0.157514967738923	0.00216605217249106	0.365444425598378	0.172292885179581	0.288825545298244
2	rs3244852	9708248	0.0372637235255608	0.100677042441695	0.0119163898944046	0.145830282578876	0.133404117163134	0.353019004199879
2	rs3312294	4407977	0.0380467913740284	0.0833527010947869	0.00833192062019866	0.0203370645268698	0.023015855778295	0.135496762142888
2	rs3386842	4408809	0.0382848811473437	0.083507380006817	0.00700649628919335	0.644909526378105	0.299808222279776	0.51008550160904
2	rs3336493	107044297	0.0382920116747383	0.0373071472448573	0.00679942521640986	0.139760779454025	0.212804236077529	0.261366211104462
2	rs3346772	5956633	0.0384072702066742	0.0121759670438374	0.00902763629746269	0.459292669797264	0.304308522050447	0.281744385446702
2	rs3331849	107117522	0.0384760925932951	0.0220822635747315	0.00477917461353888	0.0807544899571381	0.132401976508004	0.215287949203692
2	rs3463734	6195341	0.0386265973385001	0.0781205842191404	0.0113080116428823	0.219363350917563	0.266045186209608	0.404389711512285
2	rs3414813	52047699	0.0415275029900339	0.0828737225335657	0.00294446189011145	0.167081558780254	0.117105884408945	0.157727008690581
2	rs3412916	143453247	0.0423439049166624	0.0305292216582321	0.00519725764945061	0.100547004233656	0.158159406943658	0.241950145925271
2	rs3340578	145910999	0.0423439049166626	0.0305292216582322	0.0051972576494507	0.100547004233657	0.158159406943658	0.241950145925271
2	rs3398032	28860909	0.0423439049166626	0.0305292216582322	0.0051972576494507	0.100547004233657	0.158159406943658	0.241950145925271
2	rs3325427	71229194	0.0426411683785499	0.0633150541018699	0.00162544293906005	0.26485561142337	0.200932386721513	0.372918224628575
2	rs3437633	6951070	0.0451602495286489	0.0399754426967507	0.00544551342739346	0.0932240023633573	0.118833011904445	0.106503328331676
2	rs8121161	8506433	0.0473942037999439	0.04776599446793	0.0107444616435149	0.0775218054275007	0.264758222779848	0.19454930990093
2	rs1969527	8651058	0.0492595268788747	0.0398455970608279	0.0100996321963378	0.0621814169428306	0.224434409443027	0.217081696648
2	rs3222632	8504973	0.0492595268788747	0.0398455970608279	0.0100996321963378	0.0621814169428306	0.224434409443027	0.217081696648
2	rs8147459	8487074	0.0492595268788747	0.0398455970608279	0.0100996321963378	0.0621814169428306	0.224434409443027	0.217081696648
2	rs3296056	113213153	0.0501951961305051	0.364985919700095	0.00976060772523268	0.19261216419145	0.0707595221855238	0.317887858228313
2	rs3417196	8424293	0.0515414110517716	0.00679035956784541	0.00544681527726169	0.0764288254928222	0.142083736334713	0.183931378679462
2	rs3365616	6067175	0.0517812591306841	0.0362469728502878	0.00617611814582923	0.0605677111501313	0.135237840689656	0.21450442281838

2	rs3290302	35436874	0.0524309240888777	0.0503242636359191	0.0101861894214942	0.17317471537095	0.178783987316287	0.324667788362163
2	rs3397677	119309425	0.0534481716839419	0.0454183090182426	0.00856649566678947	0.0393935553551672	0.0439080215768211	0.0567214287547952
2	rs5561834	8470325	0.0550895927839252	0.0666873072620573	0.00794857885055594	0.113192728743922	0.337127334297278	0.209291485886864
2	rs8079445	140848191	0.0550960476076539	0.23002736144074	0.00154640201383483	0.462301434449887	0.232543610131101	0.368609311901224
2	rs3201139	53230161	0.0562729636468378	0.0756363682699701	0.0045066364385162	0.134358271657295	0.232928215621346	0.37498333902532
2	rs7109676	130861729	0.0584205875485014	0.412843096504391	0.00880408719888948	0.396282496777121	0.512662781020421	0.655831414256239
2	rs3339867	87188580	0.0594214821023431	0.0824052819487678	0.0118326528807308	0.0846258629206858	0.0610598458320248	0.18756632208939
2	rs3376297	139885560	0.0611454158526504	0.545956874221555	0.00349353150320965	0.465478001339993	0.323410497959009	0.440945376495234
2	rs3291678	61010117	0.0628669110452344	0.189388322598304	0.00500618038617328	0.20288652858415	0.0501355125306912	0.24280184071525
2	rs6939734	11385202	0.0628674833716746	0.0440284619396895	0.0101879084875249	0.125804094421686	0.130163966005462	0.277722593993328
2	rs3190231	58274288	0.0647443308641154	0.0494686596468204	0.0092809039242996	0.13679067418444	0.205503977530458	0.218938635331755
2	rs3355247	53071367	0.0648497350184825	0.0171030748708011	0.00792568446382544	0.0276794812794575	0.0305879997157308	0.0188878895057861
2	rs3300287	59589437	0.0657057413977188	0.212171540762784	0.00620682400106233	0.328357233944682	0.140893850686664	0.297412085533483
2	rs3313590	66667239	0.0699667573727654	0.421336689736299	0.00724208619450727	0.502866434677235	0.409694103579187	0.440426481319658
2	rs3192683	3550497	0.0785213558143473	0.0579412657042112	0.0106625309826886	0.162670897316063	0.256735177556632	0.316891008464154
2	rs3447265	29777832	0.0785213558143476	0.0579412657042113	0.0106625309826888	0.162670897316064	0.256735177556632	0.316891008464154
2	rs3185430	20011284	0.0805955421599937	0.107584782401038	0.00269255759420773	0.462767666966502	0.218157734288488	0.308915992126848
2	rs3451228	3550403	0.0832306527878684	0.134583554927299	0.00801267907901458	0.195587189812758	0.384941128182372	0.489536691137887
2	rs6911636	45085058	0.083957244484633	0.0028150498950162	0.00866652365118706	0.140628911714106	0.17388630933522	0.34472674310591
2	rs8121148	69295074	0.0895334977145609	0.59516084298682	0.0109132146968932	0.55198869782312	0.491027011773069	0.538026940570021
2	rs3191380	162099305	0.0901687919590346	0.251421280930626	0.0102539846920453	0.50261413964314	0.770202799500638	0.639603329033219
2	rs8121058	107456589	0.102662545103457	0.173064917442623	0.0686196716720211	0.00878346171417062	0.0224794317398296	0.00148242973449847
2	rs8136866	3598295	0.10916022758873	0.303173150617141	0.00521334629581156	0.429284665409667	0.0868216901183497	0.374659374659828
2	rs3284039	17968731	0.147620932193932	0.191857326414915	0.112103801085181	0.00866751755144251	0.00522298520184458	0.188215672404615
2	rs3425178	124629256	0.150612046651529	0.389023689770039	0.00797890436198457	0.440869125974598	0.0963379172010502	0.387153599001683
2	rs3292546	3762082	0.165382845252365	0.201042676343557	0.00759811390342476	0.672541367831573	0.367164922121742	0.469907733787108
2	rs3224270	10366300	0.200139689758372	0.279636934846583	0.00590842643747648	0.0325903601418099	0.758147337347863	0.207118612347629
2	rs3304077	149476133	0.200840225665928	0.172497893483474	0.00246714788546428	0.286029447250287	0.0135503364310059	0.664941979398839
2	rs3221993	2976878	0.274350033011803	0.300452909281252	0.00976205966356203	0.515353301364507	0.163823932966833	0.285634627456071
2	rs3306990	6232852	0.654432842448559	0.681123097136882	0.0103272111698876	0.21378170479874	0.405781752569283	0.49891891733606
3	rs3186073	140442770	0.00046264821910122	0.199455384163147	0.0044956366475908	0.324844423401514	0.467212947974308	0.551780447177999
3	rs3219120	51093471	0.000910600326432547	0.161901170869873	0.00704624892532969	0.0584036018699144	0.0762101214884107	0.16500846906939
3	rs3275635	75479003	0.00119700607701089	0.117647303855513	0.00143132146578057	0.228127948082776	0.291502194412751	0.374676949083734
3	rs3231662	110256290	0.00144925140938012	0.102591774219096	0.00109909390466691	0.20674478988452	0.226104851853815	0.215016188310896
3	rs3228780	54729760	0.00200461231495734	0.371318989758905	0.0110524162516829	0.0961968048617315	0.0462061490135286	0.170049840565589
3	rs1969587	49102108	0.00307079881651106	0.120663527745057	0.00322583010776098	0.0456052565081967	0.0515562746935478	0.153798117259959
3	rs3274045	34791369	0.00341196320017829	0.0592912289754704	0.00011008566082961	0.108330784120271	0.0952103934950349	0.228573968657928
3	rs3363882	9677526	0.00397319232534075	0.282103084614935	0.00893342421572834	0.452112550859205	0.611786989741745	0.706881550413126
3	rs3404732	16506568	0.00419053070755259	0.161787403866869	0.00389708924907921	0.00209905233446641	0.0351095481069384	0.124653574027981
3	rs3198722	133660770	0.00474388694294693	0.0630985729639914	0.00293464972964985	0.0942147061899654	0.0586541193308847	0.081536475608021
3	rs8121670	1168795	0.00530542760614258	0.561112171159166	0.0288799175585758	0.00147723833166294	0.0177828731356993	0.000446447210354452
3	rs3238849	90197413	0.00542146633516039	0.0638383655965715	0.00643176037640885	0.106115472442242	0.251546477560871	0.163597114674162
3	rs3430982	55033458	0.00744791644077417	0.109978672291311	0.00433338952364343	0.105689919658096	0.0339179715519011	0.249517490586401
3	rs3380475	45572627	0.0100838099699188	0.12625687260319	0.00166737068149995	0.250922884379747	0.196969669426996	0.476911914922483
3	rs8132007	9679148	0.0118082024071809	0.318198187421851	0.00545654365467187	0.212449213194588	0.0794176187422209	0.361995764851021
3	rs3319777	18750377	0.0126832809832882	0.233723385175048	0.00384588185389461	0.0724097045136388	0.259275777961203	0.275165223341059

3	rs3352361	77094749	0.0139686660007038	0.154075746765721	0.0104678955220412	0.147652374609822	0.0702952249741271	0.281726641026217
3	rs3197874	134082934	0.0143288807269363	0.0932682163431514	0.000305126909839116	0.205593216353807	0.157131701537014	0.345149642320847
3	rs3182642	60842243	0.0143886103950162	0.105977494134964	0.00655801079489506	0.104090309539945	0.0387642546875861	0.256860416650655
3	rs3359765	60842150	0.0143886103950162	0.105977494134964	0.00655801079489506	0.104090309539945	0.0387642546875861	0.256860416650655
3	rs3388802	29056946	0.0149829152978176	0.201047266917818	0.00269404743130387	0.0735976989245961	0.0927475494536353	0.221184602955021
3	rs3279352	135774892	0.0168843618815276	0.0960526749459517	0.0108877881393201	0.160837011218337	0.109602260785092	0.133691764790075
3	rs6999397	15665486	0.0168843618815276	0.0960526749459523	0.0108877881393204	0.160837011218338	0.109602260785092	0.133691764790075
3	rs3400775	6693379	0.0180247222850767	0.186542102230461	0.000673011742291235	0.0934366986234011	0.0960830545759733	0.0162406926798774
3	rs3278666	90742878	0.0195020992859059	0.00185499372391153	0.00795688322309873	0.296889510273534	0.326143540886683	0.561186789259704
3	rs3358828	135773744	0.0226516027076687	0.327161021128783	0.0321811177757073	0.00193347240209044	0.0250535311795251	0.00515697209139389
3	rs3460274	135774121	0.0281369354327813	0.0334822001252552	0.00273459287178072	0.0800759446173637	0.148143367879294	0.207424198517441
3	rs8130750	135773910	0.0281369354327813	0.0334822001252552	0.00273459287178072	0.0800759446173637	0.148143367879294	0.207424198517441
3	rs8133263	135773709	0.0281369354327813	0.0334822001252552	0.00273459287178072	0.0800759446173637	0.148143367879294	0.207424198517441
3	rs1969530	140442528	0.0289313219262311	0.130962159927636	0.00127520298749568	0.159505215414417	0.021322271230145	0.197522391856188
3	rs3188382	10945743	0.0305470631950596	0.133741599714065	0.00105405795739599	0.248602568957393	0.213936570225771	0.40524398661801
3	rs3251866	18071970	0.0341511616373727	0.105862269815812	0.00412310986642569	0.146349892160458	0.0776329790418706	0.129503242744366
3	rs3190536	120015719	0.0379832716336365	0.434533612714525	0.0107145194155869	0.14851030991038	0.115841835333656	0.0921175033992631
3	rs3317750	42010131	0.0390177402672877	0.104980344124688	0.0108129306880077	0.263777358902504	0.13147992132442	0.0562655182394795
3	rs3250734	118698604	0.039194900939575	0.426156391699873	0.00528552136665584	0.0576508174308147	0.281801036424618	0.0524100089954583
3	rs8121670	1168711	0.039952359208628	0.596226078316504	0.117131111906752	0.00184362792357279	0.00592418213831071	0.00608414364797501
3	rs7907951	74954256	0.0416534069941933	0.0115585009907637	0.00996243752139376	0.1063452075715	0.190308189561073	0.239554789872991
3	rs3298707	74994469	0.0423439049166626	0.0305292216582322	0.0051972576494507	0.100547004233657	0.158159406943658	0.241950145925271
3	rs8121511	113261912	0.0424026660824284	0.0473784362224239	0.00906070756463973	0.139815151637845	0.203823254616471	0.308681919341283
3	rs3247218	140481977	0.0430254144858091	0.0269991920171066	0.00262017886501389	0.0545574289214095	0.13333623242895	0.225611065712492
3	rs4543550	118991798	0.0458445550070332	0.444300498692909	0.0064276691844358	0.202546642720584	0.165108000897881	0.241749135731994
3	rs3279439	34791856	0.0458445550070334	0.44430049869291	0.00642766918443609	0.202546642720586	0.165108000897881	0.241749135731993
3	rs3190808	119000485	0.0497870396805122	0.222801865562031	0.000989249777550573	0.0557154787856614	0.083854936005752	0.0743074685967857
3	rs7881609	109403689	0.0501951961305049	0.364985919700094	0.00976060772523251	0.192612164191449	0.0707595221855234	0.317887858228314
3	rs3241238	43258106	0.0506737416720563	0.126376923364705	0.0110149750907565	0.0644545420209761	0.122469637548391	0.136906488962946
3	rs3227311	18708289	0.0515414110517716	0.00679035956784541	0.00544681527726169	0.0764288254928222	0.142083736334713	0.183931378679462
3	rs3268961	18086348	0.0529956496839178	0.0471202334560714	0.00866301618310661	0.131628444942535	0.187724802300558	0.2837622946128
3	rs3339759	135774503	0.0534570971279046	0.0391035547592539	0.00681306457546247	0.0255616335990216	0.0269682904498718	0.0230913269082707
3	rs3265758	119068760	0.0550960476076542	0.230027361440738	0.00154640201383481	0.462301434449888	0.232543610131101	0.368609311901224
3	rs7090787	59389077	0.05930797907805	0.0469513747642268	0.0104089509989762	0.129452249256911	0.198580155365977	0.294054174599176
3	rs3242498	16505486	0.0726604603598213	0.180575102390096	0.00411932633961813	0.223393908272786	0.186534517763461	0.402428098989452
3	rs3312610	34802427	0.073146626744139	0.0216650052146494	0.00384677280114326	0.201576139823547	0.173496590586754	0.2904579102751
3	rs3329517	34798653	0.073146626744139	0.0216650052146494	0.00384677280114326	0.201576139823547	0.173496590586754	0.2904579102751
3	rs8137611	119018106	0.0785213558143473	0.0579412657042112	0.0106625309826886	0.162670897316063	0.256735177556632	0.316891008464154
3	rs3229664	71097294	0.0924202689735382	0.470075291606267	0.00767905519111516	0.424156216852572	0.943995857909854	0.603822948419952
3	rs3218939	134081561	0.0979735073202689	0.12606293028056	0.00295813504173328	0.231777748493961	0.0458747866637891	0.128166146665249
3	rs3336028	34802866	0.099350586541331	0.0533848668666464	0.00536327825461869	0.242045383210409	0.182317433489534	0.208386902958117
3	rs3235395	135769426	0.104265621980776	0.592550083908101	0.155508310359486	0.00729056764500566	0.151544796502458	0.00309262632987667
3	rs8093947	103260466	0.105531218170771	0.264371242979245	0.00581690061433073	0.629848466584226	0.358337278666375	0.52944804103637
3	rs3213470	16660535	0.111681945657973	0.13973952095204	0.00394782907598255	0.451951921978702	0.396250377090039	0.52131796918366
3	rs3361445	16660534	0.111681945657973	0.13973952095204	0.00394782907598255	0.451951921978702	0.396250377090039	0.52131796918366
3	rs3190634	10950720	0.137733811469992	0.314568324305091	0.0028327361995034	0.285165245228656	0.122319048666718	0.326556198458623

3	rs3196775	18829833	0.151616239895891	0.20057407206482	0.00684861868159912	0.642690128127469	0.489760331727337	0.413207014740279
3	rs3389064	6049588	0.160672221418397	0.0666143558705132	0.00886345752788612	0.0733957054360968	0.0717155995033121	0.148988188103018
3	rs3329678	26191047	0.165787215909222	0.189544258581167	0.00394875554151484	0.615504248435453	0.377498497468585	0.415345838094342
3	rs3286166	17095583	0.20535564755803	0.7271792036719	0.00489317895644535	0.492492149756878	0.99330735831902	0.411188781263186
3	rs8121043	74938330	0.720496761339334	0.653976865607156	0.0107697445655916	0.395296551122657	0.306102995848486	0.0643157350936311
4	rs7043981	101913226	0.00185037573752588	0.169431722799615	0.0104567754291609	0.302197923909925	0.285263619489506	0.442374990523819
4	rs3326261	680873	0.00474388694294692	0.063098572963991	0.00293464972964982	0.0942147061899648	0.0586541193308844	0.0815364756080215
4	rs7138483	30228250	0.0064167698446831	0.0431207492046151	0.000106925419913089	0.0994977663181993	0.0915659767373804	0.232573046185213
4	rs3318567	107108170	0.00651169416270308	0.0597517032114412	0.00168416834306367	0.216984646052337	0.146999836072251	0.232580676954975
4	rs3202086	590433	0.00703909491964822	0.295954912008768	0.0109350596227603	0.220486650717964	0.0978116458160822	0.355678402950814
4	rs3256970	511359	0.00869545482497085	0.098599835923929	0.0021104954173449	0.171477132125306	0.105190823599856	0.313228456870385
4	rs8083720	119078761	0.0136373481565459	0.18687846063932	0.0080836550573264	0.0986072808403496	0.263900407409719	0.473442636802576
4	rs5561876	98616034	0.0145330460086101	0.0330160684477897	0.0101704743530925	0.446490827382867	0.258997244507257	0.538577130650196
4	rs8099121	481404	0.0156809779714261	0.0279205288135905	0.000332546012845097	0.0706299237739638	0.0199524081244673	0.287687227583418
4	rs3314837	84149019	0.0157592262481804	0.0933976954756761	0.0012207936366241	0.230335872732723	0.154980400148096	0.398944885246848
4	rs3364422	74655619	0.0192068261804972	0.221484756575596	0.00573502609398542	0.232445870834504	0.281851198070349	0.484505968948166
4	rs3369413	139804716	0.0200768178454632	0.169638062011956	0.00110567405008687	0.239622709612157	0.266801706097222	0.498222357101242
4	rs3402453	45017210	0.0281369354327813	0.0334822001252552	0.00273459287178072	0.0800759446173637	0.148143367879294	0.207424198517441
4	rs3416302	44907392	0.0281369354327813	0.0334822001252552	0.00273459287178072	0.0800759446173637	0.148143367879294	0.207424198517441
4	rs3232406	103449663	0.0304306528187477	0.307747071661373	0.00576424265962728	0.150206986783962	0.128449661622279	0.314097440089236
4	rs7913484	106283730	0.0305470631950596	0.133741599714066	0.001054057957396	0.248602568957393	0.213936570225771	0.40524398661801
4	rs3207092	107344094	0.0315633440184208	0.0381321413635935	0.00171633741255882	0.0935416253099771	0.0596748615175541	0.34657836297629
4	rs3303724	98096357	0.0322556909416104	0.121037557982462	0.00138642299384898	0.318313431489564	0.252158673483273	0.46483749328932
4	rs3244292	36670173	0.0384760925932951	0.0220822635747315	0.00477917461353888	0.0807544899571381	0.132401976508004	0.215287949203692
4	rs3406087	391557	0.0392486703378339	0.15931515540409	0.00909254202208656	0.149527292295033	0.180339767493718	0.561065319602662
4	rs3226222	107636581	0.039959943044984	0.0529409337583795	0.00926473253851777	0.154970281748909	0.0473119874591453	0.317412843156742
4	rs3383021	75048130	0.0416534069941933	0.0115585009907637	0.00996243752139376	0.1063452075715	0.190308189561073	0.239554789872991
4	rs3349468	510528	0.0423439049166626	0.0305292216582322	0.0051972576494507	0.100547004233657	0.158159406943658	0.241950145925271
4	rs3360408	121357939	0.0426411683785499	0.0633150541018699	0.00162544293906005	0.26485561142337	0.200932386721513	0.372918224628576
4	rs3406143	102235878	0.0448366262138429	0.131294311378583	0.0034534035320687	0.207687665849202	0.222321359649452	0.483547057680426
4	rs3360120	88951106	0.0456243157049062	0.0637406607470033	0.00165152582456405	0.226638052030032	0.209777056437838	0.372936108602923
4	rs8079681	90936534	0.0515449205686065	0.323740175933054	0.00234241353694961	0.300855522219975	0.511566969890236	0.608183749473693
4	rs3378803	142197626	0.0525360153682245	0.0477224018521101	0.00707061102703598	0.126310137901548	0.197482880148354	0.268897621720468
4	rs3252234	129797734	0.0534481716839419	0.0454183090182426	0.00856649566678947	0.0393935553551672	0.0439080215768211	0.0567214287547952
4	rs8090509	33354941	0.0569174134195436	0.387051243787735	0.00473019323624648	0.0911410276442106	0.0544088464382456	0.255439878650029
4	rs3427003	98096734	0.0585454248887385	0.19919522813541	0.00460714299254188	0.232223826787827	0.0477440586975311	0.105095503891817
4	rs3366013	109285951	0.0593079790780498	0.0469513747642266	0.0104089509989761	0.129452249256911	0.198580155365976	0.294054174599176
4	rs3379551	109285575	0.0593079790780498	0.0469513747642266	0.0104089509989761	0.129452249256911	0.198580155365976	0.294054174599176
4	rs3365567	102507179	0.0648762263138502	0.0113827828527588	0.0117699928466644	0.071475537557346	0.0281943130347479	0.0638983093126277
4	rs3424764	19543525	0.0684723365764723	0.0764549560424297	0.0101833770983716	0.0681995578751471	0.0366114006292126	0.0695988668198296
4	rs3459795	121552666	0.0716343689866714	0.0303275635713457	0.00857915775919123	0.0574440687349199	0.0719108088980723	0.0789716610896106
4	rs3297442	98299576	0.0726237217399503	0.0357295031786669	0.00775709507102094	0.107181364300565	0.152524084211946	0.300383331864171
4	rs3397199	90392534	0.106670630264077	0.166644466419103	0.00430350129922207	0.264049063299551	0.312846579240384	0.252103609347263
4	rs3333184	119659150	0.109499395219967	0.13673587449252	0.00649672232985041	0.256003434170617	0.189400539115005	0.641049983681394
4	rs3433266	119659648	0.109499395219967	0.13673587449252	0.00649672232985041	0.256003434170617	0.189400539115005	0.641049983681394
4	rs8087412	64744122	0.110732390401173	0.087259659668136	0.0117869005655884	0.182258731870145	0.226821988088888	0.177408614900505

4	rs3243176	87135831	0.200840225665928	0.172497893483474	0.00246714788546428	0.286029447250287	0.0135503364310059	0.664941979398839
5	rs3260018	42376816	0.000403859133365586	0.113874807986438	0.000482817504230312	0.0741716489404834	0.145714869229645	0.152190821077986
5	rs3240929	60286150	0.00207368479333776	0.0122338518728084	0.00156445174978067	0.367980032803343	0.361966233161177	0.434475401853545
5	rs8099291	12297489	0.00216964577322513	0.144706093647982	0.00434546302224354	0.00781967798080896	0.0488081709568648	0.0896759781893515
5	rs3415793	66282626	0.00223648330526575	0.174899444133457	0.00719444183487195	0.195584872828624	0.155416711784202	0.308217792486922
5	rs3248569	60987038	0.00234750790387948	0.133619440884159	0.00468708505652436	0.0672086712936497	0.0808410398175951	0.217386046682744
5	rs3406054	91689095	0.00397319232534068	0.282103084614936	0.0089334242157285	0.452112550859205	0.611786989741744	0.706881550413125
5	rs3252929	68113700	0.00435423749058112	0.156253727879725	0.00405731388015963	0.0290921868274099	0.167410473691883	0.119858109663279
5	rs3221735	93643550	0.00518010511253322	0.126472457522564	0.00453988543419882	0.038067091267044	0.0249733425825814	0.184464501670414
5	rs3259384	55261851	0.00634345540510123	0.133496740048764	0.00986035483605733	0.181378831810969	0.0813935819926274	0.138043465061549
5	rs3266913	8416027	0.00900817676074971	0.215527310303848	0.0113503377253331	0.191655146996718	0.0921008401389851	0.108837474871833
5	rs3273005	19367674	0.00985649404628159	0.0564701260322917	0.0047286900863481	0.0838819034606732	0.1353724455227	0.343436356020484
5	rs3232289	17953314	0.00985649404628162	0.0564701260322921	0.00472869008634815	0.0838819034606732	0.1353724455227	0.343436356020484
5	rs3368090	18961952	0.0122998694550656	0.319026194362888	0.0109148950087957	0.459433349368789	0.418786362974138	0.324957531035751
5	rs8094090	60975506	0.0134303833855333	0.212798877957417	0.0113901633662414	0.177668394142462	0.330283461842705	0.364728356517652
5	rs3454937	62806342	0.0140251023417634	0.284297458032763	0.0104377489522937	0.0113648194662068	0.130501463842381	0.0707134573069256
5	rs3281344	82957799	0.0147529758889048	0.0754089926608732	0.00104849606955726	0.158864433111975	0.1424810979772	0.304878966405801
5	rs3182513	16489548	0.0157826200900831	0.0859162372872122	0.00750617955193126	0.146883495325359	0.05185673163794	0.137553225862056
5	rs3423553	68020390	0.0225251537626755	0.0146308921454253	0.00467555310301955	0.153197322132427	0.0509530759431756	0.062178039468669
5	rs3264546	12378190	0.0227799054393947	0.276758994527819	0.00819034969161356	0.284446599750937	0.161468289047792	0.411154904885071
5	rs3371922	14039327	0.0235592452738063	0.0217457602939702	0.00673141028309562	0.171316215867492	0.25936364919536	0.355954476183527
5	rs3319973	68122728	0.0239820431767606	0.136024587351487	0.00803504042779756	0.183649165028304	0.0816956466571576	0.333089354800837
5	rs3316845	12453637	0.0281369354327813	0.0334822001252552	0.00273459287178072	0.0800759446173637	0.148143367879294	0.207424198517441
5	rs3323935	16354547	0.0281369354327813	0.0334822001252552	0.00273459287178072	0.0800759446173637	0.148143367879294	0.207424198517441
5	rs3342498	81901918	0.0311845898403254	0.304848557809486	0.00362223322582988	0.0820701807140629	0.0683137568965588	0.2888768699383
5	rs3192186	3357382	0.0323155459869488	0.123613607746095	0.00166136499121992	0.144118129186053	0.0547424120064801	0.211814104418707
5	rs7054011	66274582	0.0328497705880348	0.0936318999141206	0.000636518089946905	0.22976510890032	0.0985008089894595	0.281624450523232
5	rs3387381	65090470	0.0374806874173427	0.0316300364078249	0.00307822146722933	0.0716901764108491	0.141956281374733	0.175181744180643
5	rs3258692	6376143	0.0385928018835511	0.039258260801242	0.0020973483419815	0.10837089070461	0.124254950900257	0.270667243436853
5	rs3221760	55272236	0.0397885563376042	0.0467834132850337	0.007213132594605	0.0991520616524839	0.197255422710453	0.28868887725119
5	rs3405474	55271773	0.0397885563376042	0.0467834132850337	0.007213132594605	0.0991520616524839	0.197255422710453	0.28868887725119
5	rs3291876	17309796	0.0418754125006006	0.02666197721	0.00602018454187987	0.0698284542576391	0.010619834599533	0.157064072591776
5	rs3243896	93644508	0.0423439049166626	0.0305292216582322	0.0051972576494507	0.100547004233657	0.158159406943658	0.241950145925271
5	rs3248021	17870001	0.043224880492149	0.0294789792596427	0.0073879697028076	0.175876625193924	0.0942855583678047	0.358929626040556
5	rs3240288	110956592	0.0521302891077157	0.0397433852087601	0.00424896676904203	0.107175104914169	0.159486211482951	0.274377339163397
5	rs3234360	92639746	0.0554010837399413	0.0478145742030704	0.00645116877857185	0.057036220926647	0.0254213347159629	0.0662326279056783
5	rs3240881	51211105	0.0592988945349682	0.124490528935717	0.00820982050377208	0.17729301940247	0.272878715093531	0.428941133771728
5	rs3185961	81902116	0.060422174441503	0.256104308886763	0.00861447036581959	0.400844723963345	0.222181306262374	0.30712441734359
5	rs3197153	2152069	0.0639644701842049	0.138284994893882	0.000390081215843048	0.190280674708346	0.358951539758381	0.41724758778236
5	rs3292691	99160710	0.066838646974747	0.0757715303270149	0.00708367881138732	0.132400086443694	0.0585641748203112	0.485902360696464
5	rs3232427	13688232	0.0707097967856695	0.236584948252975	0.0072760982557657	0.29396126596944	0.0562195130570979	0.335494432008197
5	rs3270603	86588554	0.0711996762831538	0.0356327581745502	0.00842122133352588	0.0563029146527469	0.0332950847721576	0.0889349666641569
5	rs3401056	16202736	0.0980713251139662	0.09134825582708	0.00802285779986218	0.153159776059518	0.00529420652322318	0.442058296973017
5	rs3214435	104173578	0.102682285061533	0.292583604544019	0.00554905574304077	0.499073614174831	0.302042229200757	0.409813417276747
5	rs3272265	6786632	0.117817345064997	0.497437233792469	0.00272523922787767	0.623680165667416	0.294095711343467	0.365174941677565
5	rs3332713	6786642	0.117817345064997	0.497437233792469	0.00272523922787767	0.623680165667416	0.294095711343467	0.365174941677565

5	rs7867811	83701336	0.118960736074306	0.137547732037402	0.00495624514960878	0.153496458663537	0.107314895990873	0.580717198031398
5	rs3315300	66187557	0.200840225665928	0.172497893483474	0.00246714788546428	0.286029447250287	0.0135503364310059	0.664941979398839
6	rs3369430	71814180	0.000426189610659292	0.407029649387076	0.00936023097976101	0.0318509498774393	0.0439997900215074	0.0863991332904634
6	rs5561857	71815493	0.000426189610659292	0.407029649387076	0.00936023097976101	0.0318509498774393	0.0439997900215074	0.0863991332904634
6	rs8121136	71813847	0.000426189610659292	0.407029649387076	0.00936023097976101	0.0318509498774393	0.0439997900215074	0.0863991332904634
6	rs3431263	16590090	0.000600810195219137	0.0497747245091346	0.00808036662380973	0.511230687250215	0.506742822311746	0.594511286384433
6	rs1969534	74620803	0.000636429928848084	0.169545244263159	0.0115484167938929	0.0325585225798912	0.0321704992041463	0.0811445735684432
6	rs3188903	59794727	0.000742942459994207	0.0871693744123082	0.000628388367751545	0.116670569142834	0.0641226131019795	0.167271672002719
6	rs3455185	92530686	0.00105058768481276	0.124370170368752	0.00237919198449954	0.206537965849907	0.264439781888731	0.229187502977951
6	rs3302088	71840571	0.00144684086048055	0.319022618857816	0.00698990203629959	0.00275739492034946	0.0171919710053231	0.0580468334595251
6	rs3385245	71840507	0.00144684086048055	0.319022618857816	0.00698990203629959	0.00275739492034946	0.0171919710053231	0.0580468334595251
6	rs3443594	71836585	0.00144684086048055	0.319022618857816	0.00698990203629959	0.00275739492034946	0.0171919710053231	0.0580468334595251
6	rs5561850	71838602	0.00144684086048055	0.319022618857816	0.00698990203629959	0.00275739492034946	0.0171919710053231	0.0580468334595251
6	rs8121416	62377063	0.00157070616271392	0.122103431960341	0.00293971956293916	0.165982873533157	0.0329988021547734	0.147356122173673
6	rs3298886	83034749	0.00188964785280309	0.371527066989518	0.0108943306066109	0.115365835773583	0.135628302940455	0.285585071394999
6	rs3352208	51440330	0.00207368479333777	0.0122338518728082	0.00156445174978065	0.367980032803343	0.361966233161177	0.434475401853545
6	rs3363629	92352163	0.00216512326866459	0.0972210698440105	0.00444202756933154	0.187020831243534	0.272351386903472	0.391915664577331
6	rs3194008	83003597	0.0022531813987609	0.346448417305446	0.0101236693134921	0.112146872044359	0.147796266084358	0.27357403904959
6	rs3200349	83010616	0.0022531813987609	0.346448417305446	0.0101236693134921	0.112146872044359	0.147796266084358	0.27357403904959
6	rs3342538	70335303	0.00307079881651106	0.120663527745057	0.00322583010776098	0.0456052565081967	0.0515562746935478	0.153798117259959
6	rs3287315	17427293	0.00343602181952184	0.317458191894124	0.0085709585663455	0.141764383596104	0.173141520275845	0.304545046432779
6	rs3303297	82845098	0.00377444169768312	0.0188245285166955	0.000674461831176758	0.241973637988387	0.218279813326386	0.138319101057746
6	rs7050902	71985363	0.00385673506141385	0.222837476520886	0.00579821354744367	0.0479527404609549	0.141387922394529	0.305475545767935
6	rs3199916	17195667	0.00489554901080079	0.0565262521754657	0.00299384077860338	0.178584229354843	0.142500158903247	0.29237882673265
6	rs3281409	40711613	0.00549992523658396	0.288819823661663	0.0107186125199987	0.129488481023272	0.138866269118606	0.233284513798508
6	rs3401065	47460452	0.00700391083631364	0.40522851817683	0.0114577922095602	0.032230745033088	0.0388200299762536	0.177157855774428
6	rs1072021	40620796	0.00718766141319144	0.0400633885517837	0.00840721711862735	0.0594938530984762	0.0684085016764279	0.086904297970935
6	rs3422204	16188284	0.00966829376017402	0.186925834804142	0.00330817904870814	0.0110494877180599	0.0209222141765046	0.0486181911538035
6	rs8149698	50017992	0.00985084614978863	0.0054236981995861	0.00215541696790526	0.195755536582363	0.0245174969048473	0.187877773079618
6	rs3391659	14340000	0.0116599304382871	0.259867849476111	0.00785857976470652	0.0860824736462267	0.131015880327337	0.111682845452123
6	rs3310223	145430640	0.0117142290455397	0.119853507231991	0.000653086879621816	0.170525211986441	0.0890041738133224	0.33408525217198
6	rs8082070	154028295	0.0122998694550655	0.319026194362889	0.0109148950087957	0.459433349368789	0.418786362974139	0.324957531035751
6	rs3374733	49801652	0.0123690513707778	0.0454347723867292	0.00986875514502982	0.304083583410689	0.0592205829132901	0.140336549751768
6	rs3418521	12056665	0.012496067440665	0.144926363032903	0.00647777903256169	0.187394255841171	0.099627806862863	0.329330380489863
6	rs3367249	71571886	0.0130678611339263	0.0818354444839078	0.00414238749559603	0.0657880254738404	0.0412847137013242	0.18099736219891
6	rs3323394	64395316	0.0139686660007038	0.154075746765721	0.0104678955220412	0.147652374609822	0.0702952249741271	0.281726641026217
6	rs3245715	42884865	0.0141387618238992	0.263636827739899	0.0115727790948953	0.080178497312974	0.00246783378767049	0.168972544395595
6	rs3265507	108763002	0.0143288807269363	0.0932682163431512	0.000305126909839114	0.205593216353807	0.157131701537014	0.345149642320848
6	rs4543210	51680668	0.0143886103950162	0.105977494134964	0.00655801079489506	0.104090309539945	0.0387642546875861	0.256860416650655
6	rs3350098	27540772	0.0144603741568211	0.0762644666824233	0.00103278118332942	0.0973174845372417	0.0391403348051446	0.141890677242107
6	rs3394538	90759392	0.0145039616901224	0.0939413445078209	0.000445158154429654	0.0294984290757697	0.0063362078858113	0.0665544651487686
6	rs3410749	146147009	0.0158082256120999	0.0891364023091027	0.00475230563515266	0.095827766891431	0.0609492228036242	0.223105198875156
6	rs3207851	140385645	0.02009368247608	0.0765910895928562	0.00334976217028632	0.109543262888374	0.0769785777942434	0.240779792456654
6	rs3282830	17427886	0.02242291151679	0.214848945325011	0.00170055789713602	0.345730647299695	0.351335872994518	0.409707039446474
6	rs8121489	82340995	0.02285931069757	0.114750900115713	0.00381927936852573	0.217524048671418	0.447294156675069	0.41261907933622
6	rs3216974	44687872	0.0234073476589881	0.210599061443396	0.0114838435421427	0.0133768875083331	0.042507229235867	0.0328513719429441

6	rs3282856	38149960	0.0234688919744756	0.129303429973012	0.00117565149580007	0.239743113704556	0.196874228258187	0.399796121647329
6	rs3280778	145436771	0.0247860489943933	0.10326059840363	0.00787492806418321	0.0135966678227093	0.0556609988360528	0.0356882962473368
6	rs3258046	83126740	0.0264680031254639	0.134030298459497	0.00489345501555053	0.0752657786761146	0.124266762453784	0.280257013919391
6	rs3291553	13291432	0.0267616520415122	0.054694603555742	0.00744032598504154	0.0349111602590405	0.147005380011823	0.213118378350685
6	rs3440770	113183373	0.0273843665516431	0.166479063268634	0.00566166656213164	0.104047424164176	0.0997636029172183	0.257749338991618
6	rs3206181	48269807	0.0281369354327813	0.0334822001252552	0.00273459287178072	0.0800759446173637	0.148143367879294	0.207424198517441
6	rs3223731	93656652	0.0281369354327813	0.0334822001252552	0.00273459287178072	0.0800759446173637	0.148143367879294	0.207424198517441
6	rs3326585	37922872	0.0281369354327813	0.0334822001252552	0.00273459287178072	0.0800759446173637	0.148143367879294	0.207424198517441
6	rs3424655	69396333	0.0281369354327813	0.0334822001252552	0.00273459287178072	0.0800759446173637	0.148143367879294	0.207424198517441
6	rs3430066	69396913	0.0281369354327813	0.0334822001252552	0.00273459287178072	0.0800759446173637	0.148143367879294	0.207424198517441
6	rs3450699	68868001	0.0281369354327813	0.0334822001252552	0.00273459287178072	0.0800759446173637	0.148143367879294	0.207424198517441
6	rs3350980	78336829	0.0321322911226503	0.12210762464253	0.0102351529917759	0.0754803050680386	0.125540975273747	0.269470253846817
6	rs3257805	111005152	0.0322943762763738	0.110285129440847	0.00912225679130924	0.133454389905956	0.0930237340191553	0.352231980374589
6	rs3186748	145449816	0.0325681726215984	0.324074111970198	0.00401317868352987	0.383143460227735	0.426380778618272	0.629281520203006
6	rs8121397	156396121	0.0341084634306828	0.0985425893006107	0.00927832055307103	0.0734637676063461	0.0583188186056521	0.258502264485862
6	rs5561859	119079398	0.0341511616373727	0.105862269815812	0.00412310986642569	0.146349892160458	0.0776329790418706	0.129503242744366
6	rs3338018	77131131	0.0371444842329245	0.0822289140230916	0.0025591220231982	0.0730111645316952	0.00899737805946426	0.112020209085186
6	rs3206314	16739670	0.0374806874173427	0.0316300364078249	0.00307822146722933	0.0716901764108491	0.141956281374733	0.175181744180643
6	rs3386721	40015072	0.0375539862284073	0.0352226415131383	0.00486190177636084	0.124803866116426	0.0371348796320453	0.0163713956035057
6	rs3304170	5312062	0.0407480418551054	0.0960155027325552	0.00444502290730534	0.101771323776675	0.0925824924068689	0.112086169537077
6	rs3435166	40711610	0.0413560032213152	0.0737123776321521	0.00905808992175972	0.322032871101972	0.376266666562749	0.655596077784639
6	rs3272737	81867862	0.0416534069941933	0.0115585009907638	0.00996243752139382	0.106345207571501	0.190308189561073	0.239554789872991
6	rs3292051	143553361	0.0423129862627431	0.0350624612767419	0.00420968011870026	0.0659188529058121	0.0532550293444501	0.192980914556024
6	rs3183022	34522188	0.0423439049166624	0.030529221658232	0.00519725764945061	0.100547004233657	0.158159406943658	0.241950145925271
6	rs3361135	38010621	0.0423439049166624	0.030529221658232	0.00519725764945061	0.100547004233657	0.158159406943658	0.241950145925271
6	rs3241002	90831305	0.0423439049166626	0.0305292216582322	0.0051972576494507	0.100547004233657	0.158159406943658	0.241950145925271
6	rs3337494	137280309	0.0423439049166626	0.0305292216582322	0.0051972576494507	0.100547004233657	0.158159406943658	0.241950145925271
6	rs3349471	90829826	0.0423439049166626	0.0305292216582322	0.0051972576494507	0.100547004233657	0.158159406943658	0.241950145925271
6	rs3360202	145448885	0.0423439049166626	0.0305292216582322	0.0051972576494507	0.100547004233657	0.158159406943658	0.241950145925271
6	rs3266292	25094719	0.0442722554573604	0.165328099251881	0.000724063950022747	0.164085968083006	0.289113223713432	0.354975809487402
6	rs3342311	40025003	0.0456246881450819	0.0500961093832244	0.00774186285379385	0.142872109003118	0.207531081910706	0.29683566079241
6	rs3403421	57852280	0.0489502677721978	0.0253263717314747	0.00985724218876107	0.145002333746555	0.224286069033868	0.331790249423866
6	rs3297702	104384185	0.0492595268788747	0.0398455970608279	0.0100996321963378	0.0621814169428306	0.224434409443027	0.217081696648
6	rs3205456	14649938	0.0501951961305051	0.364985919700094	0.00976060772523263	0.19261216419145	0.0707595221855241	0.317887858228313
6	rs3257593	141230791	0.0505281385370427	0.318269177341164	0.00145394623540314	0.0921401733861979	0.101172361422515	0.0720108330080456
6	rs3446279	48213063	0.0524124377940403	0.0177731995752471	0.0110205632488151	0.0245260425833747	0.13699016817935	0.183918296127482
6	rs3442946	90781658	0.0524916952828347	0.03331250803127	0.00893284860702472	0.102428041825769	0.164747921309111	0.260604584507048
6	rs3254637	54382137	0.0527523974348899	0.131734794399077	0.00349545544336907	0.323275420563996	0.0108439721954363	0.299865450908317
6	rs8121086	16567311	0.0551513127841433	0.0405664435419737	0.00120457303111184	0.158313093517728	0.0365380754012724	0.239729080460855
6	rs3380418	145415425	0.0582262129245834	0.135560236984669	0.00170003817340566	0.0998138517074317	0.0139043953524471	0.190225740484043
6	rs3432361	155180452	0.0584205875485015	0.412843096504391	0.00880408719888951	0.396282496777121	0.512662781020422	0.655831414256239
6	rs3446221	145432538	0.0596500175104572	0.0500823805721316	0.00759837605459784	0.196166862998233	0.257302866447435	0.40919779570916
6	rs3453406	4216676	0.0628669110452345	0.189388322598304	0.00500618038617336	0.202886528584151	0.0501355125306913	0.24280184071525
6	rs3232235	12710247	0.0684651254507353	0.0369127711554234	0.00732271281701228	0.063070454509679	0.0487986764187748	0.0860632722896531
6	rs3275805	12056361	0.0698490022378171	0.0230592840455141	0.0115966244847497	0.154114799415033	0.193363626396146	0.341114502046823
6	rs5561926	81869984	0.0714331130592373	0.0425785278327702	0.00967316540695786	0.133433858403078	0.22139747923303	0.285092725444293

6	rs3315291	111623624	0.0714662456892575	0.0658896422531099	0.00459289103088497	0.977369433129221	0.59645784938219	0.764426179564521
6	rs3375237	42902751	0.0781853747465358	0.0417137977487229	0.0112802203627097	0.31132188119136	0.201991971438229	0.402459176604888
6	rs3271016	92051495	0.0785213558143473	0.0579412657042112	0.0106625309826886	0.162670897316063	0.256735177556632	0.316891008464154
6	rs8121289	73129339	0.0785213558143476	0.0579412657042113	0.0106625309826887	0.162670897316064	0.256735177556632	0.316891008464154
6	rs3402920	18007118	0.0825798891640415	0.130745241307976	0.00932696555329437	0.503996319248578	0.412900554297323	0.539202096658263
6	rs3382564	145450588	0.0863623232492014	0.00799521961073149	0.0102162104968229	0.165088727578612	0.238322853748593	0.3157336181445
6	rs3374469	45127264	0.0880494558726699	0.0594118125364206	0.0107263882884708	0.196146586284531	0.293790928885554	0.408524535294538
6	rs3236535	77348591	0.0951666569316175	0.309040492286656	0.00505581720557285	0.306736855361041	0.219632890964805	0.68617311095134
6	rs8121856	23714643	0.0979735073202691	0.12606293028056	0.00295813504173337	0.231777748493963	0.0458747866637894	0.128166146665249
6	rs3427957	12242162	0.104265621980776	0.5925500839081	0.155508310359487	0.00729056764500566	0.151544796502458	0.00309262632987666
6	rs3185396	152539284	0.106552413048522	0.185172497309879	0.00685576160978756	0.114229448959528	0.0361559340482251	0.213655443173903
6	rs3408694	4473767	0.108019884325931	0.0924540775371951	0.00607427614213815	0.276375443150793	0.0449715087062334	0.37248958241577
6	rs3305202	53298177	0.10916022758873	0.30317315061714	0.00521334629581153	0.42928466540967	0.0868216901183488	0.374659374659828
6	rs3338204	71511833	0.116957790573574	0.648506376753561	0.0117998287140315	0.0354528471869374	0.218695140607907	0.171402599964901
6	rs3389686	134913501	0.117194520170357	0.181703644879933	0.00792143690585063	0.39248703478603	0.144285093231961	0.339057306208321
6	rs3370208	155340806	0.124196799809035	0.51868744117301	0.0107596382028973	0.103261581762071	0.278524984680196	0.243424619930044
6	rs3221042	141388208	0.12688447498836	0.121290439828212	0.00368474252736588	0.251378440528001	0.0599572266300806	0.422454880414405
6	rs3281074	155180863	0.145926987903056	0.363409663725667	0.00929578322080775	0.160489546319724	0.138934138818463	0.0496909286745996
6	rs3273767	154050379	0.274350033011803	0.300452909281252	0.00976205966356232	0.515353301364508	0.163823932966832	0.285634627456072
6	rs3447828	77203835	0.281456188139147	0.706158620662174	0.00240709314863607	0.424112564387182	0.564816060742509	0.666634110413573
6	rs3351849	5141032	0.615622217232209	0.165552291115996	0.000419642148041214	0.560688021516316	0.0396180133788311	0.876139434099243
6	rs3282641	141543733	0.720496761339335	0.653976865607154	0.0107697445655904	0.395296551122662	0.306102995848484	0.0643157350936327
7	rs3207913	48167285	0.00049418590366574	0.0442731022504209	0.000255349768038171	0.0952094938500478	0.12832272047956	0.188930582330368
7	rs3247976	14559378	0.000600810195219209	0.0497747245091339	0.00808036662380944	0.511230687250214	0.506742822311746	0.594511286384434
7	rs3194442	43488686	0.000865105741409581	0.186885295322831	0.00860542616744473	0.0847541094400136	0.183492971021325	0.327404114483373
7	rs3352797	43488649	0.000865105741409581	0.186885295322831	0.00860542616744473	0.0847541094400136	0.183492971021325	0.327404114483373
7	rs3351221	39244687	0.00300092963884998	0.102805372068004	0.00635578638655458	0.237614068761293	0.046629766521711	0.332085786463857
7	rs3273363	43381113	0.00306254987624909	0.082843990734524	0.0105284299013377	0.166838524575885	0.356908130399334	0.418038090700523
7	rs3298533	34988786	0.00372151927463825	0.0196924115748562	0.00101368450806551	0.210094943531612	0.108103511637149	0.358182629082989
7	rs3221092	26956005	0.00847167087359107	0.24537183453734	0.0114009678950628	0.00899931642834717	0.0359732257969207	0.0781014753564317
7	rs3418833	60145787	0.00900817676074971	0.215527310303848	0.0113503377253332	0.191655146996718	0.092100840138985	0.108837474871835
7	rs4543156	26944896	0.0105423618780452	0.101301748559874	0.0115366104812956	0.0419866566943119	0.0874317869741427	0.14270911772428
7	rs3344364	50147776	0.0137758134004138	0.229106511341943	0.0078129351842324	0.0176524563097507	0.0378197023994903	0.0291077139158124
7	rs3339039	2048636	0.0171387467852789	0.208716703553595	0.00512186716322155	0.103773536020519	0.0997602690099982	0.229193940260208
7	rs3293039	43489263	0.017778762337844	0.234818674926101	0.00917384764943042	0.0285879952608593	0.0809744526475702	0.0250429680161596
7	rs7062927	48148406	0.0233247293287754	0.0960302511344066	0.00930116941622356	0.100094378809166	0.0362045913773977	0.126774269236619
7	rs3449043	5313125	0.0247354947194507	0.0143242072125587	0.00578601127940731	0.244217554622882	0.268648115272985	0.250093022801771
7	rs7069317	22545762	0.0262496250638064	0.0152879585799422	0.00518632132210136	0.0817029100852417	0.174657862939988	0.199870049127405
7	rs3443670	26934403	0.0273116482881819	0.0304068767793127	0.00101692651745404	0.0761529250219778	0.0945106392687725	0.214952797733935
7	rs3218876	69923603	0.0281369354327813	0.0334822001252552	0.00273459287178072	0.0800759446173637	0.148143367879294	0.207424198517441
7	rs3386937	26716321	0.0289313219262311	0.130962159927636	0.00127520298749568	0.159505215414417	0.021322271230145	0.197522391856188
7	rs3206237	48302254	0.0305511572115682	0.242730351449853	0.00616479787691406	0.102475187255201	0.126630418070983	0.309569531611924
7	rs3243723	48302700	0.0305511572115682	0.242730351449853	0.00616479787691406	0.102475187255201	0.126630418070983	0.309569531611924
7	rs3299314	48301865	0.0305511572115682	0.242730351449853	0.00616479787691406	0.102475187255201	0.126630418070983	0.309569531611924
7	rs3300797	48301770	0.0305511572115682	0.242730351449853	0.00616479787691406	0.102475187255201	0.126630418070983	0.309569531611924
7	rs3312579	48683267	0.0305511572115682	0.242730351449853	0.00616479787691406	0.102475187255201	0.126630418070983	0.309569531611924

7	rs3341890	48702834	0.0305511572115682	0.242730351449853	0.00616479787691406	0.102475187255201	0.126630418070983	0.309569531611924
7	rs3355181	48679808	0.0305511572115682	0.242730351449853	0.00616479787691406	0.102475187255201	0.126630418070983	0.309569531611924
7	rs3394379	48679528	0.0305511572115682	0.242730351449853	0.00616479787691406	0.102475187255201	0.126630418070983	0.309569531611924
7	rs3400083	48301777	0.0305511572115682	0.242730351449853	0.00616479787691406	0.102475187255201	0.126630418070983	0.309569531611924
7	rs3419683	48302896	0.0305511572115682	0.242730351449853	0.00616479787691406	0.102475187255201	0.126630418070983	0.309569531611924
7	rs3450706	48148191	0.0312038445697374	0.0766479631596543	0.00167866282053985	0.113446493986761	0.00364389692620755	0.226899781339499
7	rs3330967	34139052	0.032548980699728	0.259946096789521	0.00575579567406904	0.312248240701616	0.310264000632618	0.164434648964626
7	rs3385325	27723100	0.0333964716172245	0.0295402098984687	0.00789511093400068	0.131771930063165	0.180514123675543	0.281724118561053
7	rs3261642	83499676	0.0370778252356937	0.0483447581070779	0.00393578186074217	0.144725511847866	0.189748275975309	0.293462644116993
7	rs3243846	34159557	0.0383732230922472	0.00986075594435934	0.0107112233065036	0.0254742071225032	0.0687316195578368	0.195033579421093
7	rs3187631	26674524	0.0384989191581018	0.158334429884226	0.00651374515012988	0.220863629868409	0.01525149748365	0.22821576470133
7	rs6996410	3379381	0.0422246543989796	0.0596987567543704	0.00957287836768103	0.134033739894687	0.162766082831112	0.291019849261645
7	rs3385918	83021324	0.0423439049166626	0.0305292216582322	0.0051972576494507	0.100547004233657	0.158159406943658	0.241950145925271
7	rs3452857	29490473	0.0423439049166626	0.0305292216582322	0.0051972576494507	0.100547004233657	0.158159406943658	0.241950145925271
7	rs3203194	39021117	0.048644579725352	0.163843177541755	0.00224746508908245	0.461879346197824	0.320635715785472	0.579450153206223
7	rs3460920	45119851	0.0501951961305049	0.364985919700094	0.00976060772523251	0.192612164191449	0.0707595221855234	0.317887858228314
7	rs3317900	53312588	0.0503065802008086	0.0443298075299265	0.00708297855853052	0.13630886642086	0.200001779111119	0.260346854535767
7	rs3411698	164571	0.0510106976824026	0.121857357236951	0.00719096088984983	0.18081813586625	0.171670516604897	0.604287495069675
7	rs8098986	24817635	0.0527523974348899	0.131734794399077	0.00349545544336907	0.323275420563996	0.0108439721954363	0.299865450908317
7	rs3274195	14642240	0.0534481716839414	0.0454183090182425	0.0085664956667894	0.0393935553551667	0.0439080215768208	0.0567214287547951
7	rs3348875	48119706	0.0550960476076542	0.230027361440738	0.00154640201383482	0.462301434449888	0.232543610131101	0.368609311901224
7	rs3416029	83565184	0.0559046028827726	0.0510189170690959	0.00402197279485976	0.137960402455141	0.197438424930666	0.263677704217599
7	rs3268831	29179813	0.0582262129245834	0.135560236984669	0.00170003817340566	0.0998138517074317	0.0139043953524471	0.190225740484043
7	rs3451960	48119733	0.0586730771668969	0.109574955424403	0.00263557583719043	0.126094756846528	0.0226010583689447	0.244192865861403
7	rs7895489	12889502	0.0593079790780498	0.0469513747642266	0.0104089509989761	0.129452249256911	0.198580155365976	0.294054174599176
7	rs3364304	39254889	0.0599076161847083	0.0386748919027193	0.00844436169525681	0.127010164718971	0.218891681799556	0.27001764870492
7	rs3211475	34273946	0.0601384312871527	0.0493290774608907	0.00607287849388855	0.076501298987657	0.0661747872573279	0.352036005940515
7	rs3278689	73129138	0.0612769579968838	0.0687213289146143	0.00522611983018175	0.125084677001417	0.0395191860871034	0.0189578467360237
7	rs6912341	45103099	0.062491062296256	0.0240020381868494	0.00387546136428229	0.0557176201846661	0.0191929316122268	0.0833673520532846
7	rs3412879	27716349	0.0678532655769085	0.0146925674297649	0.0117379384627832	0.0636799823204162	0.0514996063310187	0.109737943496289
7	rs3379302	43492696	0.0701470618344268	0.0150726059357071	0.00978102080494694	0.0180430067112363	0.0983307013784149	0.117269185520622
7	rs8094509	43526977	0.0701470618344268	0.0150726059357071	0.00978102080494694	0.0180430067112363	0.0983307013784149	0.117269185520622
7	rs8079728	43492620	0.070147061834427	0.0150726059357071	0.00978102080494697	0.0180430067112363	0.0983307013784151	0.117269185520622
7	rs3380517	83499712	0.0812238968541305	0.236680328173204	0.0103103367673732	0.181244928288291	0.228862519948055	0.369639454006458
7	rs1071993	83456587	0.0850947174449569	0.305186368539204	0.00350970876916144	0.349939106668155	0.0723361105606238	0.437598961976733
7	rs3382361	27986368	0.0930072924592136	0.0414084313859985	0.00586873405247668	0.164046228708889	0.0965402383306578	0.41880097361513
7	rs3280714	1784251	0.0939456293774722	0.258515003510153	0.00546379067036335	0.35656309272086	0.0767681416154591	0.0681358055297702
7	rs3296040	33117119	0.112554699218017	0.101429607669527	0.011376177484178	0.253614943540077	0.202810974238103	0.328081443962162
7	rs3300787	27567531	0.114321523165386	0.0361497890464097	0.00966329298025029	0.202630388236668	0.268913206938348	0.2615245899997
7	rs3353160	27602815	0.115525617421159	0.226237975400558	0.00525660467133448	0.108417361809107	0.131447204075315	0.202514430955498
7	rs3422675	24818070	0.141902558539361	0.172384166894976	0.00696272018492925	0.353544315825356	0.136576362913159	0.205051675353249
7	rs3343584	24117546	0.274350033011803	0.300452909281252	0.00976205966356217	0.515353301364509	0.163823932966832	0.285634627456071
7	rs3321848	14563442	0.756622732848534	0.19005358646771	0.0019935994792331	0.272904096658073	0.956612975104325	0.31609110162821
10	rs3218929	71227207	0.00527102828440784	0.0239766535893001	0.0029946104941769	0.075654449861034	0.0860769912905731	0.248735187592743
10	rs3384928	35067331	0.00707466197979936	0.526255444918104	0.0105186592785239	0.0275264948603564	0.0555223848180175	0.257643729306482
10	rs3348051	29217609	0.00726369716801976	0.0533117240319091	0.000316020169650435	0.135115261816779	0.104570561208607	0.268966200316102

10	rs3189779	61652537	0.0089172527597436	0.1693928004128	0.00176184953734942	0.172814551728678	0.21381041579428	0.400675688129151
10	rs3396714	52884599	0.0100173383182429	0.100613892950313	0.00934063926297526	0.121067382744463	0.262901553916252	0.354444907483253
10	rs3449888	15053131	0.0101497993578925	0.0370812128098592	0.000239005375768452	0.102232900641036	0.0618156566215356	0.25288846587492
10	rs3257384	29520123	0.0122998694550659	0.319026194362887	0.0109148950087953	0.459433349368788	0.418786362974138	0.32495753103575
10	rs3241382	14937143	0.0127557627050202	0.0493150756391057	0.00197229463126039	0.151028582184514	0.10235227657056	0.2342795888793
10	rs3361492	14937171	0.0127557627050202	0.0493150756391057	0.00197229463126039	0.151028582184514	0.10235227657056	0.2342795888793
10	rs3369775	65735350	0.0139686660007038	0.154075746765721	0.0104678955220412	0.147652374609822	0.0702952249741271	0.281726641026217
10	rs3268991	28397620	0.0139686660007038	0.154075746765722	0.0104678955220412	0.147652374609823	0.0702952249741266	0.281726641026216
10	rs3339725	18750591	0.0143288807269363	0.0932682163431512	0.000305126909839114	0.205593216353807	0.157131701537014	0.345149642320848
10	rs3222225	65807958	0.0171387467852789	0.208716703553595	0.00512186716322155	0.103773536020519	0.0997602690099982	0.229193940260208
10	rs3395807	47235383	0.0182415018496304	0.182854577314135	0.000778787749655973	0.230765025792444	0.311667841695452	0.439422513582524
10	rs3307710	16759791	0.0187603726690894	0.0293753493854075	0.000651880562790478	0.0785857285376566	0.0282054690334361	0.266213003286606
10	rs3295339	21916161	0.0188712371296138	0.395548818001198	0.0101255616414295	0.389256386835963	0.164797938254059	0.268184661300686
10	rs3299982	38205963	0.0190220478818779	0.0814883227934527	0.0026284655192081	0.387880498681907	0.418934529748714	0.62400876515185
10	rs3391149	48960256	0.0223363284575019	0.0341170075188855	0.000356343382840642	0.197997021447175	0.142647534001136	0.291586260919258
10	rs3194821	61452526	0.0275796200185951	0.727163368230843	0.0111422095739297	0.274484607108584	0.310355925984846	0.4404649385256
10	rs3413832	12288033	0.0281369354327813	0.0334822001252552	0.00273459287178072	0.0800759446173637	0.148143367879294	0.207424198517441
10	rs3458380	24606321	0.0281369354327813	0.0334822001252552	0.00273459287178072	0.0800759446173637	0.148143367879294	0.207424198517441
10	rs4543040	16021616	0.028448072336869	0.254646107121536	0.00604921746266042	0.0865630856307416	0.144854285500087	0.251074569812767
10	rs8121813	29289496	0.0289313219262311	0.130962159927636	0.00127520298749568	0.159505215414417	0.021322271230145	0.197522391856187
10	rs3449843	56131102	0.0295902906644405	0.1082009641437	0.00357230990764316	0.118357461495387	0.0670267087556335	0.157134067439611
10	rs7009757	47234866	0.0381910747228294	0.104116671730659	0.0109476065559976	0.0879856605998176	0.207830754320399	0.269284795569687
10	rs3417672	24616331	0.0405279977195073	0.0260730701631047	0.0104071807563014	0.0995413306901122	0.215363132901609	0.238865420733693
10	rs8121160	37312422	0.0409397542289342	0.0458613718085365	0.00617423922003983	0.125906935730731	0.194054289974879	0.256585187758177
10	rs3194656	33123563	0.0458445550070329	0.444300498692909	0.00642766918443527	0.202546642720582	0.165108000897881	0.241749135731994
10	rs3183050	12318247	0.0515414110517716	0.00679035956784541	0.00544681527726169	0.0764288254928222	0.142083736334713	0.183931378679462
10	rs3430213	12275757	0.0515414110517716	0.00679035956784541	0.00544681527726169	0.0764288254928222	0.142083736334713	0.183931378679462
10	rs8142931	12516174	0.0515414110517716	0.00679035956784541	0.00544681527726169	0.0764288254928222	0.142083736334713	0.183931378679462
10	rs3243317	12259644	0.0516163379034001	0.0253401728218007	0.00896872704146589	0.0919545476109958	0.176038404815709	0.180777415170251
10	rs3234761	28234008	0.0534481716839419	0.0454183090182426	0.00856649566678947	0.0393935553551672	0.0439080215768211	0.0567214287547952
10	rs3243639	71209484	0.0550960476076538	0.230027361440741	0.00154640201383486	0.462301434449888	0.232543610131102	0.368609311901224
10	rs8121813	29289436	0.0550960476076542	0.230027361440738	0.00154640201383482	0.462301434449887	0.232543610131101	0.368609311901224
10	rs3341154	52885811	0.0589113358761958	0.0567300463006107	0.00141261935073568	0.251408809328441	0.0289414096519793	0.339630525701166
10	rs3208443	47055880	0.0628669110452342	0.189388322598304	0.00500618038617324	0.202886528584149	0.050135512530691	0.242801840715251
10	rs3352864	18266237	0.0657057413977186	0.212171540762785	0.00620682400106228	0.32835723394468	0.140893850686663	0.297412085533483
10	rs3446864	13570900	0.0722065745842442	0.245211664717632	0.0100752039616176	0.14593218204932	0.474906091195991	0.338009106445158
10	rs3421265	29915350	0.0828478926619924	0.0883041651564973	0.0117300557760055	0.0610739313765388	0.0604463553038947	0.139432304723292
10	rs3438988	17111931	0.08953349771456	0.595160842986821	0.0109132146968929	0.551988697823114	0.491027011773063	0.538026940570016
10	rs3316880	63659407	0.0895334977145609	0.59516084298682	0.0109132146968932	0.55198869782312	0.491027011773069	0.538026940570021
10	rs3226856	52884772	0.720496761339334	0.653976865607155	0.0107697445655902	0.395296551122661	0.306102995848487	0.0643157350936313
11	rs3378183	23598085	0.000600810195219209	0.0497747245091339	0.00808036662380944	0.511230687250214	0.506742822311746	0.594511286384434
11	rs3283312	86584930	0.0029916450634229	0.0499545751388017	0.00071753256048593	0.153377302824897	0.254564213265959	0.434690038565698
11	rs6905511	16000653	0.00398573926996396	0.073915916651581	0.00435571792117824	0.00426156011606779	0.0265583357409099	0.087128966496507
11	rs3463337	16001432	0.00658561784762909	0.24612229275519	0.00577649587864779	0.172338644149333	0.0458404219275824	0.244065293163741
11	rs3430671	22983721	0.0139686660007038	0.154075746765721	0.0104678955220412	0.147652374609822	0.0702952249741269	0.281726641026217
11	rs6955334	84410623	0.0139686660007038	0.154075746765721	0.0104678955220412	0.147652374609822	0.0702952249741269	0.281726641026217

11	rs3449221	49591324	0.0189322115243488	0.226104815670564	0.00106996352920775	0.128761255706759	0.129931016966983	0.287620715020853
11	rs3367301	84430618	0.0207147127269841	0.398978242852886	0.00392204791223662	0.0897934544956558	0.107375553200132	0.251862462317142
11	rs3249517	72182983	0.0382823633971459	0.184684862851634	0.0103657034141617	0.0321727809265718	0.132747692635056	0.0569019952688678
11	rs3378415	15547041	0.0385928018835511	0.039258260801242	0.0020973483419815	0.10837089070461	0.124254950900257	0.270667243436853
11	rs3448639	13200489	0.0570384543653061	0.82560138669323	0.00989640251998851	0.395579850084292	0.450969622119291	0.698178903032404
11	rs3427941	84425045	0.0575405166772773	0.297441484161707	0.00130693761280844	0.361004845512349	0.10885357064203	0.290958074175211
11	rs3326893	83558514	0.0757722175843392	0.0394597503191056	0.010510440872434	0.0300216756682192	0.0241993913497556	0.0806033276668889
11	rs3389066	8641206	0.141902558539361	0.172384166894976	0.00696272018492905	0.353544315825357	0.136576362913159	0.20505167535325
12	rs8126097	21057842	0.00269864329876018	0.115008453263899	0.00591333284505199	0.156729361219237	0.0536271059539532	0.135431038857718
12	rs3321622	18430488	0.00289019515628126	0.169041273618502	0.00483959967660752	0.352376087624721	0.163133617762847	0.251523472840703
12	rs8121163	21054282	0.00293678666104732	0.214044132063643	0.00324901486455011	0.433886687612577	0.316985363705378	0.311245218249973
12	rs3189696	20427345	0.003170388142862	0.0570458858391232	0.00152660268656138	0.0570152219138726	0.0245814277332984	0.14541761596016
12	rs8121145	23969774	0.00459823146411799	0.289641363776296	0.0073203614799776	0.10913363816094	0.0713597861826559	0.239343453090078
12	rs3230303	1614758	0.00474388694294692	0.063098572963991	0.00293464972964983	0.0942147061899648	0.0586541193308844	0.0815364756080211
12	rs3426051	21008281	0.00511811103564341	0.167216474674265	0.00191113316135942	0.0425974080715475	0.0353904945896026	0.102785104539919
12	rs3444973	27633518	0.00715028737540831	0.0855617456966358	0.00124547200231988	0.247367006506817	0.310537307101251	0.45989271943532
12	rs3379898	55218746	0.0090401140130236	0.205289665192086	0.00459128199958514	0.361026400023593	0.294360944448912	0.242411544256994
12	rs3429928	62500771	0.0110924807133239	0.38152519348617	0.00736717489046361	0.149664061293691	0.442528692997093	0.657488521230425
12	rs3264645	14070119	0.0122998694550656	0.319026194362889	0.0109148950087956	0.459433349368788	0.418786362974139	0.324957531035751
12	rs3265015	16904270	0.0123690513707778	0.0454347723867292	0.00986875514502982	0.304083583410689	0.0592205829132901	0.140336549751768
12	rs7011088	20347069	0.0123690513707779	0.0454347723867294	0.00986875514502984	0.30408358341069	0.0592205829132901	0.140336549751768
12	rs3334126	49737749	0.0123721858252966	0.0341377094490213	0.00316825812964556	0.0645279709888548	0.0927693947188141	0.266336431458624
12	rs3307345	18310242	0.0124752927550246	0.0523046238435983	0.00144412504227871	0.192566520493036	0.175384607937267	0.253266179755378
12	rs3377202	14661877	0.0130332382622422	0.11383820643198	0.00541113097713924	0.142563107160947	0.148939348730586	0.3158645509435
12	rs3326340	26406250	0.0135287000890122	0.0791969168786385	0.000769783872109122	0.171076513543848	0.138560500076766	0.311610913998798
12	rs3340256	62669028	0.0139686660007038	0.154075746765721	0.0104678955220412	0.147652374609822	0.0702952249741271	0.281726641026217
12	rs3336204	62667823	0.0143886103950162	0.105977494134964	0.00655801079489506	0.104090309539945	0.0387642546875861	0.256860416650655
12	rs3226490	49682575	0.0145803379652084	0.217768393173981	0.00576356316748183	0.0708461273926868	0.0958088071469202	0.327005210035351
12	rs3269613	18310861	0.0151745856694042	0.128232176383587	0.00166062357307522	0.10100211239459	0.140823238827441	0.166197535782221
12	rs3340368	20463247	0.0184440424994286	0.115873883947784	0.0013271357969865	0.181063211926016	0.209785948418064	0.398123662334083
12	rs3273510	54990249	0.0194823977119945	0.0301922102182299	0.0102048048415568	0.637597672978981	0.489063989064286	0.435647227336306
12	rs3410473	26406384	0.0211092387258506	0.0407223308347357	0.00308454605414309	0.0236825246932021	0.11021347717403	0.184297592954022
12	rs3215524	21280934	0.0234073476589882	0.210599061443396	0.0114838435421427	0.0133768875083331	0.042507229235867	0.032851371942944
12	rs3377621	4628217	0.0293149675243153	0.0662002973664014	0.000877694537335861	0.197893327956056	0.192065717793282	0.380784554988005
12	rs3356089	20247657	0.0294853919371986	0.0951640149960953	0.00684799268168592	0.0230384413702044	0.0534956523733169	0.073291756860693
12	rs3432031	57413445	0.0305511572115682	0.242730351449853	0.00616479787691409	0.102475187255201	0.126630418070983	0.309569531611924
12	rs3277908	57895614	0.0347221447325521	0.405456560941841	0.00882540589847648	0.140866304979835	0.123899092611302	0.338279467434534
12	rs8121333	57411427	0.0348670803628745	0.277377574922028	0.00735822920932682	0.00806762346851782	0.0329837443099851	0.146984890049233
12	rs3375897	5641783	0.0351860087293135	0.0280954517774762	0.00799175221585278	0.122566077450912	0.189313820324853	0.212478860884008
12	rs3284105	58069564	0.0390430832504373	0.112201184387281	0.00885809851630263	0.0137840505667177	0.0900221871425568	0.145674267528225
12	rs3421589	26359795	0.0392486703378339	0.15931515540409	0.00909254202208656	0.149527292295033	0.180339767493718	0.561065319602662
12	rs3281677	52120842	0.0423439049166624	0.0305292216582321	0.00519725764945061	0.100547004233656	0.158159406943658	0.241950145925271
12	rs3299788	49900886	0.0423439049166626	0.0305292216582322	0.0051972576494507	0.100547004233657	0.158159406943658	0.241950145925271
12	rs1969531	6455366	0.0429074509595119	0.00924126574957136	0.0104403888081755	0.382997380383807	0.395838550009598	0.453860361486676
12	rs1969576	6455357	0.0429074509595119	0.00924126574957136	0.0104403888081755	0.382997380383807	0.395838550009598	0.453860361486676
12	rs3261036	40083713	0.0461196123746731	0.236775596898578	0.00252245189910434	0.571177681807094	0.489102531598555	0.632791057875296

12	rs3409408	53966755	0.0501417103072301	0.0650880189630588	0.0110855634813187	0.149670123787678	0.265276112167557	0.349403482778131
12	rs3277368	41711487	0.0507600712482943	0.0872802310764549	0.00439067387304673	0.205110245941313	0.024859243522357	0.273220179130507
12	rs3383773	31729216	0.0586785873580505	0.188350742790788	0.0380237624818242	0.00390199396319151	0.00823805137347202	0.0409945447449151
12	rs3350718	8000086	0.0593079790780498	0.0469513747642266	0.0104089509989761	0.129452249256911	0.198580155365976	0.294054174599176
12	rs8147794	20454434	0.0610785033902774	0.0343573148679931	0.00977006730795662	0.094309640422393	0.1384222585538	0.277648283886708
12	rs3272916	53986383	0.0785213558143476	0.0579412657042113	0.0106625309826888	0.162670897316064	0.256735177556632	0.316891008464154
12	rs8144089	17124761	0.0786879683969808	0.0200369934477419	0.00658776481779931	0.0622191967558519	0.00234723418465798	0.100553462981442
12	rs3385944	15294939	0.086298386112371	0.0326139304213435	0.0050284028102855	0.155821687141888	0.18593073654187	0.290759472300092
12	rs3420381	46529091	0.0964338019238798	0.070879485413975	0.0109697532267288	0.206431096346623	0.244452906121095	0.277594614819416
12	rs3315845	40714061	0.108875109771773	0.282562095801702	0.00205860655010136	0.13869063427556	0.137931806018001	0.0299528886087258
12	rs3335796	62500763	0.110288691076882	0.135856443577609	0.00942528616186593	0.210243638558321	0.0175836715251498	0.540292693533708
12	rs3349424	75937	0.115249003483641	0.131086406731356	0.00419105049308714	0.396175761292702	0.328289565760042	0.359641319499559
12	rs3291076	55567965	0.283475669470564	0.293197206417267	0.0103355490993723	0.899467850942391	0.680807224014435	0.754399307702321
13	rs3302927	23695323	0.00151977032511054	0.0345419364740519	0.000788674329678462	0.126323685792635	0.145253622387328	0.23487383797612
13	rs3378701	170594040	0.00151977032511054	0.0345419364740519	0.000788674329678462	0.126323685792635	0.145253622387328	0.23487383797612
13	rs3235848	108078945	0.00301702286532553	0.0400503028057997	0.0038733369036709	0.252431961953409	0.288211063609022	0.336193677023128
13	rs3202535	73202125	0.00471996332819965	0.0628297563032198	0.00262305986983059	0.174807944758563	0.180538968182664	0.293205898109932
13	rs3198425	2572889	0.00726369716801977	0.0533117240319092	0.00031602016965044	0.13511526181678	0.104570561208607	0.268966200316102
13	rs3286285	12220534	0.00972957423498168	0.0869364423017083	0.000670731090372508	0.190854866441647	0.156206400019363	0.298076270358364
13	rs3433261	12220022	0.00972957423498168	0.0869364423017083	0.000670731090372508	0.190854866441647	0.156206400019363	0.298076270358364
13	rs3365955	81357569	0.00985649404628159	0.0564701260322917	0.0047286900863481	0.0838819034606732	0.1353724455227	0.343436356020484
13	rs8097959	34564939	0.00991044282642287	0.0983696726740425	0.00476878371837048	0.0346230810893803	0.15761632564387	0.265601498232543
13	rs3412895	24844900	0.0139686660007038	0.154075746765721	0.0104678955220412	0.147652374609822	0.0702952249741271	0.281726641026217
13	rs8093275	25179377	0.0139686660007038	0.154075746765721	0.0104678955220412	0.147652374609823	0.0702952249741265	0.281726641026216
13	rs3289111	90385316	0.0194463072751358	0.098367889971265	0.00125457603632987	0.141939438837048	0.137154892292116	0.349691355341811
13	rs3338873	210482884	0.024264322198402	0.0281448698930664	0.00745906134316106	0.0928340098021603	0.159809102587768	0.16502036834331
13	rs3279163	20618054	0.0260095355839012	0.309524662203798	0.00269416811464922	0.283413799182942	0.391187631404994	0.521032974511899
13	rs3421724	7996770	0.0281369354327813	0.0334822001252552	0.00273459287178072	0.0800759446173637	0.148143367879294	0.207424198517441
13	rs8147869	32216343	0.0295263994084431	0.0799947911827154	0.00124774432273342	0.0908058446368309	0.0258666663017211	0.189843940455048
13	rs3375370	32579634	0.0313265468294337	0.120882801125899	0.00376844533399659	0.148104749005783	0.055700585347279	0.0628566749372273
13	rs3276294	8040312	0.0313784008722854	0.0631117094755706	0.00137402013667754	0.221695030149799	0.110759606479921	0.418524070557324
13	rs3244809	192508154	0.0339343359610853	0.116135661454502	0.00670341076940153	0.173958067318217	0.124540161028405	0.360392250320878
13	rs3303022	131632781	0.0339529609991467	0.0424172907695233	0.00195952352429172	0.101621153727965	0.138853661347673	0.265805413850186
13	rs3220111	199291797	0.0343126549884863	0.051426549746508	0.00704330275292788	0.0700538337192394	0.105762461806802	0.0813408192830586
13	rs3461621	60423087	0.0405355850341412	0.120591656797152	0.0107889692177248	0.110022038699009	0.295431792841355	0.332652123813657
13	rs3412680	31640209	0.0418660593323715	0.0621698724335227	0.0106759422872593	0.101820629849354	0.0816388587031291	0.196314115761568
13	rs3366212	3626929	0.0423439049166626	0.0305292216582322	0.0051972576494507	0.100547004233657	0.158159406943658	0.241950145925271
13	rs8121361	21237277	0.0477627588314052	0.0600141854676125	0.00994354618566223	0.134494611928334	0.263855330750764	0.352530275537511
13	rs3209578	26130238	0.0496052301838711	0.123064009319771	0.0113799782594731	0.380798124105741	0.158676380774518	0.238853808144575
13	rs3377834	36909993	0.0501951961305051	0.364985919700094	0.00976060772523263	0.19261216419145	0.0707595221855237	0.317887858228313
13	rs3451173	108079632	0.0541552043817406	0.296966450693976	0.00476402038434566	0.309453372681937	0.523684315757902	0.569194814067941
13	rs8121666	91528973	0.0550960476076538	0.230027361440741	0.00154640201383486	0.462301434449888	0.232543610131102	0.368609311901224
13	rs3463585	26047648	0.0570415406964746	0.131443814044769	0.00701819325239083	0.125842988967702	0.185860263380066	0.348745133599718
13	rs3423508	96978107	0.0577097640022059	0.056769048052723	0.0042260695985461	0.2388839630642	0.387242776666952	0.543340734553636
13	rs3407175	12131329	0.0600019916163142	0.644321478092218	0.00773211967019874	0.0634494303445717	0.329871160016322	0.0340796992615578
13	rs3450422	206231976	0.0600111138997565	0.0678399041163552	0.0109380459177759	0.0625908146946894	0.0625904590601389	0.0176429731099197

13	rs3406563	26046609	0.0657057413977185	0.212171540762785	0.00620682400106229	0.328357233944682	0.140893850686664	0.297412085533483
13	rs3325869	217504973	0.0680180759631521	0.0764859354304192	0.00574345640712734	0.173342541464736	0.230625366376832	0.236666395435851
13	rs3269032	20310175	0.0684651254507353	0.0369127711554234	0.00732271281701228	0.063070454509679	0.0487986764187748	0.0860632722896531
13	rs3195677	212930351	0.0714331130592373	0.0425785278327702	0.00967316540695786	0.133433858403078	0.22139747923303	0.285092725444293
13	rs3458974	20309785	0.0861690516782402	0.0521177140271778	0.011588181545764	0.125566062008831	0.0382865169381826	0.119570066511916
13	rs8085979	35934708	0.110288691076882	0.135856443577608	0.00942528616186583	0.210243638558321	0.0175836715251499	0.540292693533706
13	rs3186945	210484468	0.142890184722774	0.0986617220003299	0.00748464092535553	0.455827236621427	0.526327201041246	0.647913671562992
13	rs3291212	210484774	0.142890184722774	0.0986617220003299	0.00748464092535553	0.455827236621427	0.526327201041246	0.647913671562992
13	rs3242923	199361600	0.144655494175811	0.0512129403687314	0.00826729441994129	0.169388723552382	0.0532161029772002	0.284747900411245
13	rs3412445	199306436	0.144655494175811	0.0512129403687314	0.00826729441994129	0.169388723552382	0.0532161029772002	0.284747900411245
13	rs3272612	206120076	0.144655494175813	0.0512129403687308	0.00826729441994148	0.169388723552384	0.0532161029771997	0.284747900411247
13	rs5561914	207340084	0.145278990764225	0.143032729204445	0.00759098203532224	0.124276537160778	0.0257712253016214	0.149422109759406
13	rs3274174	73608501	0.183969093967197	0.138416788493396	0.0114539163847162	0.288111936826201	0.440697999515146	0.393593376896755
13	rs3346266	199291655	0.376776079083791	0.36412747887279	0.00229666294822575	0.388330048682073	0.0353379990889853	0.332931996805906
13	rs3421895	28863621	0.720496761339334	0.653976865607155	0.0107697445655903	0.395296551122661	0.306102995848487	0.0643157350936314
14	rs3218264	49434505	0.000742942459994207	0.0871693744123082	0.000628388367751545	0.116670569142834	0.0641226131019795	0.167271672002719
14	rs3438132	6823063	0.000887991925764021	0.0355231880268987	0.00409604513788395	0.0919746593091762	0.206961585264044	0.455131730606471
14	rs3288774	65323473	0.00163625903449378	0.0582998402378043	0.000895042706398031	0.307219899162801	0.288727842144033	0.359335832353911
14	rs8081021	64065519	0.00169545231355578	0.045745410207621	0.000634671296413431	0.0982488039206009	0.11882815102207	0.251183405519712
14	rs3260562	143697147	0.00475045275899215	0.0885854231458667	0.0113358789380636	0.439014410722081	0.551276744659792	0.639535823966946
14	rs8089627	120072061	0.00572895959588916	0.0560581786207445	0.000499633441139312	0.245809668551144	0.19666033302536	0.443736192859079
14	rs3188714	124190799	0.0063486322778502	0.0132063602439814	0.00348874837587329	0.265777770395719	0.114826807088807	0.27704900342722
14	rs3329610	33829075	0.00726369716801976	0.0533117240319091	0.000316020169650435	0.135115261816779	0.104570561208607	0.268966200316102
14	rs3425110	78291644	0.00764253672485518	0.17957262868775	0.000868167612314121	0.0445178449668642	0.0237050866627917	0.0472731705745316
14	rs3216015	33703897	0.00815637230239739	0.206088125769011	0.00194197747840692	0.263582316782933	0.237150329642302	0.191039751974867
14	rs3230488	41287689	0.0121787749359123	0.0300530591448541	0.000920439629268903	0.158956833616379	0.141803444264383	0.2344221207291
14	rs3303860	53394297	0.0122998694550656	0.319026194362888	0.0109148950087957	0.459433349368788	0.418786362974139	0.324957531035751
14	rs3276304	118624361	0.0126774920484823	0.0682106148218433	0.00117202964093897	0.0128116098536887	0.0280186171343635	0.0432443982320957
14	rs3245875	99354470	0.0139686660007038	0.154075746765721	0.0104678955220412	0.147652374609822	0.0702952249741271	0.281726641026217
14	rs3439327	92530709	0.0139686660007038	0.154075746765722	0.0104678955220412	0.147652374609823	0.0702952249741266	0.281726641026216
14	rs6987637	122330777	0.0145527947566053	0.0288640214229266	0.00371756596064713	0.214624692905241	0.199436982553887	0.349642004289109
14	rs3294188	27802425	0.0147529758889048	0.0754089926608732	0.00104849606955726	0.158864433111975	0.1424810979772	0.304878966405801
14	rs3364679	50866845	0.016474491798131	0.0710603425156858	0.00344879454605868	0.124910716768252	0.0680441381901031	0.186662983261136
14	rs3217080	116531687	0.0168843618815278	0.0960526749459507	0.0108877881393199	0.160837011218336	0.109602260785092	0.133691764790076
14	rs3291563	140604230	0.0182415018496304	0.182854577314135	0.000778787749655973	0.230765025792444	0.311667841695452	0.439422513582524
14	rs8145066	41110559	0.0186286624674901	0.0694225207876703	0.00468586622567569	0.21910925504409	0.301697780600473	0.490663290276386
14	rs6915781	68154165	0.0216432067402465	0.12186772240119	0.0109726551519421	0.189504402819799	0.0990763043987766	0.336377093670752
14	rs7902023	52274805	0.0234073476589882	0.210599061443395	0.0114838435421427	0.0133768875083331	0.042507229235867	0.032851371942944
14	rs8080907	120071962	0.0239339665946083	0.0691303044166821	0.0056045826926454	0.178615358021515	0.0796788252913504	0.0666663513895059
14	rs3406143	140571709	0.0273339546295444	0.0619756618525178	0.000468444875920213	0.206592568138646	0.19904646064084	0.359440184999533
14	rs3283940	12846088	0.0274343786332352	0.14350785049485	0.00362235484577054	0.29300231691867	0.251267128706693	0.454554370751718
14	rs1969545	45896335	0.0281369354327813	0.0334822001252552	0.00273459287178072	0.0800759446173635	0.148143367879294	0.207424198517442
14	rs6970597	150607244	0.0281369354327813	0.0334822001252552	0.00273459287178072	0.0800759446173635	0.148143367879294	0.207424198517442
14	rs3199421	33279638	0.0281369354327813	0.0334822001252552	0.00273459287178072	0.0800759446173637	0.148143367879294	0.207424198517441
14	rs3289949	33830714	0.0281369354327813	0.0334822001252552	0.00273459287178072	0.0800759446173637	0.148143367879294	0.207424198517441
14	rs3340819	32599436	0.0281369354327813	0.0334822001252552	0.00273459287178072	0.0800759446173637	0.148143367879294	0.207424198517441

14	rs3361623	32712936	0.0281369354327813	0.0334822001252552	0.00273459287178072	0.0800759446173637	0.148143367879294	0.207424198517441
14	rs3369216	32712449	0.0281369354327813	0.0334822001252552	0.00273459287178072	0.0800759446173637	0.148143367879294	0.207424198517441
14	rs3410384	33733150	0.0281369354327813	0.0334822001252552	0.00273459287178072	0.0800759446173637	0.148143367879294	0.207424198517441
14	rs7929599	33229125	0.0281369354327813	0.0334822001252552	0.00273459287178072	0.0800759446173637	0.148143367879294	0.207424198517441
14	rs3348102	12845906	0.0287208580412675	0.235979234951103	0.00329348477417747	0.432426919589463	0.253815870748089	0.342475942931107
14	rs3462899	12845957	0.0287208580412675	0.235979234951103	0.00329348477417747	0.432426919589463	0.253815870748089	0.342475942931107
14	rs3420252	88501898	0.0320903980581091	0.0723526178610075	0.00645480974449373	0.136345669654891	0.247593844972407	0.457722497059509
14	rs8087521	118346487	0.032103773118404	0.00297755357672347	0.00493502124311874	0.0720054540559973	0.107186351552106	0.0906370270531052
14	rs3423405	36414743	0.0325681726215985	0.324074111970199	0.00401317868352992	0.383143460227735	0.426380778618272	0.629281520203005
14	rs3278261	64775214	0.0326180406184516	0.157514967738924	0.00216605217249105	0.365444425598378	0.172292885179582	0.288825545298245
14	rs3400261	84024156	0.0348835570032713	0.166470243923997	0.000538303004353968	0.948584862633815	0.726660130048775	0.974447901859865
14	rs3456358	11510384	0.0368258089085569	0.0335529197711763	0.00784015664020747	0.160281393917462	0.0353991619723792	0.254623348494965
14	rs3294336	77503458	0.0376717481163328	0.113716525440782	0.00435871816017188	0.272362376208511	0.216902920741475	0.490160199286997
14	rs3244566	36436405	0.0384760925932951	0.0220822635747315	0.00477917461353888	0.0807544899571381	0.132401976508004	0.215287949203692
14	rs3260275	32711746	0.0384760925932951	0.0220822635747315	0.00477917461353888	0.0807544899571381	0.132401976508004	0.215287949203692
14	rs3276138	32739036	0.0384760925932951	0.0220822635747315	0.00477917461353888	0.0807544899571381	0.132401976508004	0.215287949203692
14	rs3412390	31150321	0.0384760925932951	0.0220822635747315	0.00477917461353888	0.0807544899571381	0.132401976508004	0.215287949203692
14	rs3436430	33158738	0.0384760925932951	0.0220822635747315	0.00477917461353888	0.0807544899571381	0.132401976508004	0.215287949203692
14	rs3284340	81253412	0.0385593539751064	0.297845667077482	0.00488749297842794	0.0260688700488816	0.205508898252446	0.135402877262974
14	rs3204234	98575861	0.0395902815454012	0.0594787587108732	0.00114890876414794	0.128318114313658	0.0387647619740167	0.0880494812095132
14	rs3349998	34177558	0.0423439049166626	0.0305292216582322	0.0051972576494507	0.100547004233657	0.158159406943658	0.241950145925271
14	rs3461884	113508261	0.0423439049166626	0.0305292216582322	0.0051972576494507	0.100547004233657	0.158159406943658	0.241950145925271
14	rs8091352	144178016	0.0423439049166626	0.0305292216582322	0.0051972576494507	0.100547004233657	0.158159406943658	0.241950145925271
14	rs3330297	143120560	0.0427037049778332	0.0505269156416695	0.00337096283887062	0.103759357028454	0.139433744652291	0.288760765756151
14	rs3183683	140571634	0.0443317661331489	0.109190280902451	0.000609248577343864	0.00966838195190979	0.0688859106220393	0.131692769187193
14	rs8084061	143109491	0.0445336355262127	0.00893783462563298	0.0104102257046336	0.00600397320633227	0.00660465262613099	0.0346049447811447
14	rs8097134	135752181	0.0482990227219953	0.0320523904879801	0.00767692624460276	0.0291915749333493	0.0321212624019256	0.0502100978728863
14	rs3284199	98578470	0.0516075282116875	0.0634430955912895	0.00611659037811679	0.133687781240064	0.0389800499779409	0.062389017293962
14	rs3405878	145503117	0.0628669110452344	0.189388322598304	0.00500618038617328	0.20288652858415	0.0501355125306912	0.24280184071525
14	rs3257750	145502695	0.0628924610342696	0.0377772795240923	0.00997378091691978	0.132000238682807	0.1974452842305	0.288562330520408
14	rs3403333	78520462	0.0637187374282877	0.0356879875462786	0.00101411267288314	0.102644710666777	0.182673863503118	0.559074592743944
14	rs3236198	120352109	0.0748739208182478	0.0512641960468292	0.0090850904509611	0.0486988754145662	0.302489734651586	0.463899189958638
14	rs3238920	65306790	0.086256448939375	0.229589303901783	0.00453320643585834	0.110957105613925	0.094856659483786	0.119510092651868
14	rs3272195	41128934	0.0873587354513145	0.289537033044749	0.00995106493515815	0.872286713102274	0.324628468556326	0.861139286547617
14	rs3305591	32817797	0.109160227588731	0.30317315061714	0.00521334629581154	0.429284665409671	0.0868216901183489	0.374659374659829
14	rs3330681	97822263	0.111681945657973	0.13973952095204	0.00394782907598238	0.4519519219787	0.396250377090039	0.521317969183662
14	rs3207716	141053655	0.111681945657973	0.13973952095204	0.00394782907598243	0.451951921978701	0.396250377090039	0.521317969183662
14	rs3303340	21928756	0.116617023869974	0.232295050953034	0.000911585011770241	0.327191241396368	0.168487229212838	0.184638405502339
14	rs3215234	6955456	0.134113612077824	0.170745511951432	0.0105722055958877	0.532106415761617	0.29771556086445	0.378441348617436
14	rs3243733	58498506	0.471853340470488	0.178533566931876	0.000973363666425727	0.606385264505482	0.00827771191355381	0.900610826695934
14	rs3341328	78283546	0.720496761339334	0.653976865607155	0.01076974456559	0.39529655112266	0.306102995848487	0.0643157350936312
15	rs3222728	147313589	0.00207368479333776	0.0122338518728086	0.00156445174978072	0.367980032803344	0.361966233161177	0.434475401853544
15	rs3346957	85027196	0.00600386630229397	0.0129368778035915	0.00273163570659132	0.150676004425826	0.131766338330679	0.136074901012837
15	rs3382211	92427236	0.0089034231490415	0.245771081240869	0.00258963191298307	0.0977065743969209	0.0608075730753756	0.193979040512661
15	rs3374707	117591252	0.0139686660007038	0.154075746765721	0.0104678955220412	0.147652374609822	0.0702952249741271	0.281726641026217
15	rs3262132	59486901	0.014460374156821	0.0762644666824232	0.00103278118332941	0.0973174845372415	0.0391403348051445	0.141890677242108

15	rs3203639	133367799	0.0168843618815278	0.0960526749459507	0.0108877881393199	0.160837011218336	0.109602260785092	0.133691764790076
15	rs3316956	133367825	0.0168843618815278	0.0960526749459507	0.0108877881393199	0.160837011218336	0.109602260785092	0.133691764790076
15	rs3227815	153950251	0.0223620767613721	0.173441290625713	0.00166399649716032	0.436901387805591	0.308026101917547	0.640362008621839
15	rs3279596	150857956	0.0235446470945567	0.0287404758798914	0.000540984266270978	0.356153148478526	0.531498917636717	0.713093087970178
15	rs3378378	150857845	0.023544647094557	0.0287404758798913	0.000540984266270709	0.356153148478527	0.531498917636717	0.713093087970178
15	rs3456090	34660079	0.0251256269545563	0.0594337895875268	0.00555366741965993	0.0441775400349829	0.0183074070400647	0.206723770338945
15	rs3345305	88506046	0.0260095355839013	0.309524662203799	0.00269416811464929	0.283413799182943	0.391187631404995	0.521032974511898
15	rs3358740	8578207	0.032618040618452	0.157514967738922	0.00216605217249105	0.365444425598378	0.172292885179581	0.288825545298244
15	rs3355054	62266283	0.0362560707708525	0.0282610602828655	0.00816425167713466	0.178463612866269	0.322337540653441	0.369184698004752
15	rs3273259	150857719	0.0370754890371047	0.0065949106537771	0.00708290339700994	0.358131555743209	0.265843635272708	0.690412077160898
15	rs3341047	34655413	0.0405442362988152	0.0889243702514323	0.00445307156445923	0.188276658029139	0.282985862208194	0.455429261666618
15	rs3443350	34655511	0.0405442362988152	0.0889243702514323	0.00445307156445923	0.188276658029139	0.282985862208194	0.455429261666618
15	rs8145366	1931345	0.041261818303451	0.0129489619508119	0.00694205838494492	0.108534764846567	0.171875811065724	0.278820200770991
15	rs3250777	156939264	0.0423439049166624	0.0305292216582321	0.00519725764945061	0.100547004233656	0.158159406943658	0.241950145925271
15	rs3309509	55742776	0.0423439049166626	0.0305292216582322	0.0051972576494507	0.100547004233657	0.158159406943658	0.241950145925271
15	rs3301223	154436750	0.0501951961305052	0.364985919700095	0.00976060772523265	0.19261216419145	0.0707595221855238	0.317887858228313
15	rs7010029	59811305	0.0534481716839419	0.0454183090182426	0.00856649566678947	0.0393935553551672	0.0439080215768211	0.0567214287547952
15	rs3306753	34951479	0.0537664379418645	0.00727261573911662	0.00278148706244348	0.0457887463957871	0.0379913138395884	0.212695653249749
15	rs3197040	87454963	0.0584205875485017	0.412843096504393	0.00880408719888988	0.396282496777123	0.512662781020422	0.655831414256238
15	rs3235359	156947563	0.0627493786453103	0.10791016129773	0.00941841440337097	0.135264572483218	0.140906247042127	0.317452635094478
15	rs3299338	55551181	0.0771938058751988	0.237512721853702	0.00601559903347051	0.284402197821095	0.290918515997563	0.641590066119837
15	rs3381792	62269890	0.0785213558143473	0.0579412657042112	0.0106625309826886	0.162670897316063	0.256735177556632	0.316891008464154
15	rs3382770	134435750	0.0785213558143473	0.0579412657042112	0.0106625309826886	0.162670897316063	0.256735177556632	0.316891008464154
15	rs3214111	134279537	0.0801126765481016	0.107632224346474	0.00543937499417737	0.192912840285415	0.071540923811617	0.14403865705608
15	rs8092787	152266762	0.0804439865810184	0.0755388460969892	0.00488260111090777	0.16099726709238	0.155647912358889	0.179628808295479
15	rs3334480	29056823	0.0842863189679623	0.0640888035518868	0.0031138940489227	0.287908583786379	0.220969944172733	0.355598438800233
15	rs3373650	91393259	0.109659689207876	0.0684962882152247	0.0112299993096966	0.0308136125344204	0.0772961455226757	0.0906487311244957
15	rs3256904	26822438	0.110288691076882	0.135856443577608	0.00942528616186582	0.210243638558321	0.0175836715251499	0.540292693533708
16	rs3183332	81583302	0.007131449903345	0.127635963155837	0.00798558782004365	0.157049247015699	0.0998472212358099	0.0387815918870622
16	rs3437374	51705860	0.0134417876127264	0.582397316810987	0.00515753262087335	0.258086931777987	0.105520497080482	0.429004974674132
16	rs3317583	68557359	0.0186882468638167	0.0460029460646416	0.00620980752366929	0.0450962477254488	0.0794565150783544	0.025418331456789
16	rs3361784	78053316	0.0233181849292502	0.0742377757875972	0.00145728806353879	0.0753765217628244	0.130231266107381	0.257782419928273
16	rs3240413	77975877	0.0321322911226503	0.12210762464253	0.0102351529917759	0.0754803050680386	0.125540975273747	0.269470253846817
16	rs3278275	36553272	0.0324306397291394	0.0847716150723628	0.00857002993012651	0.110670211563234	0.122209329208948	0.323232256148168
16	rs7010524	78196160	0.0340021553718574	0.0319000552577648	0.00429127337274457	0.0615258595704286	0.117385354494912	0.243850104171981
16	rs3208814	82445863	0.0347949627418334	0.131576428312407	0.0119136709466106	0.271751047957633	0.488690106073352	0.490503629439932
16	rs3424767	44284232	0.0423439049166626	0.0305292216582322	0.0051972576494507	0.100547004233657	0.158159406943658	0.241950145925271
16	rs3335638	4222669	0.0581129627381979	0.201530252106604	0.00562530426377138	0.373156050384555	0.223227669003948	0.280781800928044
16	rs3271901	58081919	0.0588854930779914	0.203843711469919	0.00442300964084003	0.407068962277831	0.217457907149703	0.332381790591334
16	rs3220273	20792895	0.0832632694143644	0.0271495205972757	0.0021860943906238	0.167482117786212	0.114987938849886	0.434657311898818
16	rs3366773	46609601	0.117351187577509	0.0573872141126819	0.0116907111489937	0.138348654644084	0.000812900851629944	0.127552340825657
16	rs3312615	74250026	0.126154481177872	0.262049835615445	0.00835614281559738	0.350140732273378	0.161644000602218	0.363281758350178
16	rs3304363	78052369	0.12688447498836	0.121290439828212	0.00368474252736588	0.251378440528001	0.0599572266300806	0.422454880414405
16	rs3415126	78052420	0.16578721590922	0.189544258581168	0.0039487555415148	0.615504248435452	0.377498497468587	0.41534583809434
16	rs6974379	81669092	0.720496761339334	0.653976865607155	0.0107697445655903	0.395296551122661	0.306102995848487	0.0643157350936314
16	rs3335427	24931343	0.720496761339334	0.653976865607156	0.0107697445655934	0.395296551122662	0.306102995848487	0.0643157350936305

16	rs3342625	80366642	2.8042816017563e-05	0.454812669533891	0.00760461580697955	0.0827780981790688	0.0593864191773424	0.168329653606403
17	rs3212516	64062748	0.000278587310515823	0.0684593264535083	0.000504507871045886	0.200909394000381	0.212554647346323	0.325474651499858
17	rs3415163	66131137	0.00397319232534075	0.282103084614935	0.00893342421572834	0.452112550859205	0.611786989741745	0.706881550413126
17	rs8121119	43733590	0.00420352638569506	0.375834342049776	0.0307579954641491	0.0657024920971061	0.00333636870474715	0.270770701403242
17	rs3349904	12814784	0.00569525559640175	0.201238576369941	0.011308185883491	0.230813559359394	0.388806398942012	0.487854528799739
17	rs3373710	53452738	0.00726369716801976	0.0533117240319091	0.000316020169650435	0.135115261816779	0.104570561208607	0.268966200316102
17	rs3427347	43511147	0.00900817676074971	0.215527310303848	0.0113503377253333	0.191655146996718	0.0921008401389852	0.108837474871834
17	rs3405944	53453560	0.0093532230745937	0.136129762522744	0.00567153823162924	0.330313856536424	0.242904846274215	0.218051678538774
17	rs8099074	64089303	0.0123690513707778	0.0454347723867294	0.00986875514502993	0.30408358341069	0.0592205829132902	0.140336549751768
17	rs3351185	36230454	0.0139686660007038	0.154075746765721	0.0104678955220412	0.147652374609822	0.0702952249741269	0.281726641026217
17	rs3429858	53453320	0.0139686660007038	0.154075746765721	0.0104678955220412	0.147652374609822	0.0702952249741271	0.281726641026217
17	rs3249013	53452983	0.0139686660007038	0.154075746765722	0.0104678955220412	0.147652374609823	0.0702952249741266	0.281726641026216
17	rs3282052	40368140	0.0159563884126414	0.114014565107281	0.0111800349168051	0.0809360995320093	0.0965255142476027	0.125479542591604
17	rs3436981	42694444	0.0181784200029421	0.156398659001672	0.00445354367782672	0.255870485215709	0.16029408936312	0.3995459780445
17	rs3215396	53453597	0.0182415018496304	0.182854577314135	0.000778787749655973	0.230765025792444	0.311667841695452	0.439422513582524
17	rs3222284	59951609	0.019264559654856	0.0774678327838138	0.00605387150659371	0.0663859486441068	0.0121431639033145	0.284738222957901
17	rs3413435	36198457	0.0195551178450957	0.169359293845257	0.00161494395241262	0.323808964960616	0.25991823053633	0.361095611038189
17	rs3278536	13101910	0.0200627625062261	0.0944501755366836	0.00812302866954837	0.0612156853758992	0.0336006548880493	0.254086371705151
17	rs3436563	66541484	0.0280371513397935	0.130507174725558	0.00821362671762282	0.0652854064274122	0.0153527831387947	0.194114147571852
17	rs6943281	52813426	0.0281369354327813	0.0334822001252552	0.00273459287178072	0.0800759446173637	0.148143367879294	0.207424198517441
17	rs3279785	59378159	0.0298893292794024	0.116636853858933	0.00352707649704133	0.140285388470659	0.0641703573290095	0.185753155101746
17	rs3252915	6401346	0.0305470631950596	0.133741599714066	0.00105405795739599	0.248602568957393	0.213936570225772	0.40524398661801
17	rs3404827	66541221	0.0327312519909901	0.190592088620258	0.0119914062025006	0.0685416035284752	0.0307578251861447	0.20717894182848
17	rs1969494	52501364	0.0330457900746737	0.00864019733682116	0.00183254728755598	0.0371293419766096	0.0543986661465393	0.163703301404889
17	rs3319048	52465663	0.0357969373531825	0.0612641686964653	0.00349752000829767	0.0619688464999446	0.0784966653296938	0.226104926372477
17	rs3385386	52465695	0.0357969373531825	0.0612641686964653	0.00349752000829767	0.0619688464999446	0.0784966653296938	0.226104926372477
17	rs3330547	52814899	0.0384760925932951	0.0220822635747315	0.00477917461353888	0.0807544899571381	0.132401976508004	0.215287949203692
17	rs3385767	52813990	0.0384760925932951	0.0220822635747315	0.00477917461353888	0.0807544899571381	0.132401976508004	0.215287949203692
17	rs3398395	53228798	0.0384760925932951	0.0220822635747315	0.00477917461353888	0.0807544899571381	0.132401976508004	0.215287949203692
17	rs3409602	52814367	0.0384760925932951	0.0220822635747315	0.00477917461353888	0.0807544899571381	0.132401976508004	0.215287949203692
17	rs8078856	52808733	0.0384760925932951	0.0220822635747315	0.00477917461353888	0.0807544899571381	0.132401976508004	0.215287949203692
17	rs3424700	59401169	0.0416534069941933	0.0115585009907638	0.00996243752139382	0.106345207571501	0.190308189561073	0.239554789872991
17	rs3276935	53453351	0.0423439049166626	0.0305292216582322	0.0051972576494507	0.100547004233657	0.158159406943658	0.241950145925271
17	rs3355565	65160083	0.0458445550070329	0.444300498692909	0.00642766918443527	0.202546642720582	0.165108000897881	0.241749135731994
17	rs3437205	40367914	0.048644579725352	0.163843177541755	0.00224746508908245	0.461879346197824	0.320635715785472	0.579450153206223
17	rs3222488	52501225	0.0492079542364637	0.0201162925460716	0.00827785932698745	0.0427628332670249	0.030682973521169	0.181910119027774
17	rs3368590	64196175	0.0534481716839419	0.0454183090182426	0.00856649566678947	0.0393935553551672	0.0439080215768211	0.0567214287547952
17	rs3299084	48699916	0.0554190087523153	0.00471122785327156	0.0102176288936877	0.187730988567726	0.0213141455842895	0.223826585736584
17	rs8085055	65777816	0.0570106899307711	0.0471197341778463	0.00988345649789664	0.0502823695593361	0.104189100976413	0.0657650909337919
17	rs3302151	28116546	0.0571514112397521	0.0497995584978317	0.0109951552914751	0.147031151352171	0.219800019684988	0.311663792163025
17	rs3407101	66131575	0.0618045549016308	0.0914637540944496	0.00380738204733947	0.339894288554183	0.224095900781807	0.412746514473526
17	rs3388912	44648131	0.0729659687045385	0.0487597699807703	0.0115188115525886	0.135088800230153	0.226597463290249	0.346367691616635
17	rs3247390	45271578	0.0782505941854104	0.0211270936102411	0.0115784817480079	0.0654606810740792	0.100342890556448	0.334080616688354
17	rs3398363	15510187	0.097783034091836	0.0867514407577637	0.0111586182113628	0.0748613552651568	0.0440479152187451	0.0920686782140264
17	rs3418054	46628902	0.110288691076882	0.135856443577608	0.00942528616186583	0.210243638558321	0.0175836715251499	0.540292693533706
17	rs3198035	69088478	0.124196799809038	0.518687441173007	0.0107596382028986	0.103261581762069	0.278524984680198	0.243424619930042

17	rs3221175	57850552	0.135007115006717	0.270170535578131	0.00462524406167176	0.194327194684456	0.0906127959695235	0.325547832197833
17	rs3275855	13101805	0.15250642943524	0.0639248002966431	0.00818765137139261	0.251563118934923	0.196380338230153	0.570954557853637
17	rs3370465	57850070	0.325093106417137	0.34733960600692	0.00626080849765352	0.0540532622304214	0.157931287091294	0.944704082549937
18	rs3370523	31747058	0.00832238100538566	0.313824550339977	0.010656587097006	0.110410854435232	0.0935357147741872	0.252022115990552
18	rs3278775	40220837	0.0113164858065877	0.267953776589088	0.00764810787992655	0.121562143385392	0.136823098177041	0.0448320379443383
18	rs3222181	27260679	0.0170834396007723	0.0877319371620278	0.00312220331214368	0.117817883133987	0.0665762894892225	0.177699181188724
18	rs3416263	27260422	0.0170834396007723	0.0877319371620278	0.00312220331214368	0.117817883133987	0.0665762894892225	0.177699181188724
18	rs3431268	31746137	0.0221290339505971	0.0518289687135241	0.00204713271359889	0.109345342803828	0.0974431533488039	0.203276645609993
18	rs3251718	8555922	0.0281369354327813	0.0334822001252552	0.00273459287178072	0.0800759446173637	0.148143367879294	0.207424198517441
18	rs3219084	59921692	0.0341859666511615	0.144470903440553	0.0101771572711047	0.139489612963285	0.204122910061221	0.270560393683985
18	rs3349501	9675669	0.0416534069941933	0.0115585009907638	0.00996243752139382	0.106345207571501	0.190308189561073	0.239554789872991
18	rs3343867	9570006	0.0423439049166626	0.0305292216582322	0.0051972576494507	0.100547004233657	0.158159406943658	0.241950145925271
18	rs3394199	9675344	0.0423439049166626	0.0305292216582322	0.0051972576494507	0.100547004233657	0.158159406943658	0.241950145925271
18	rs3381150	53448987	0.0534481716839416	0.0454183090182426	0.00856649566678943	0.0393935553551669	0.0439080215768209	0.056721428754795
18	rs8121262	15608381	0.0739580284982004	0.0893179867531159	0.0103140439877885	0.237196055075597	0.115520453227423	0.11015402584984
18	rs3372206	60729725	0.08953349771456	0.595160842986821	0.0109132146968929	0.551988697823114	0.491027011773063	0.538026940570016
18	rs3400294	26638779	0.101592763604473	0.335070199273565	0.00575328912138008	0.590135882968925	0.368773262010532	0.450396449213221

Table S2. Localization of differentially expressed genes in QTL regions associated with percentage of all fiber type.

Type I muscle fibers				Type IIB muscle fibers					Type IIA muscle fibers			
**Gene**	**SSC**	**QTLs ID**		**Gene**	**SSC**	**QTLs ID**			**Gene**	**SSC**	**QTLs ID**	
PRPF39		4021		*CXCL14*		7040			*CEP162*		7015	
SAMD4A		4021		*PCDH12*		7040			*GJA1*	1	7015	
TMEM260	1	4021		*PROB1*	2	7040			*HSF2*		7015	
TMEM38B			2794	*RAD50*		7040			*SNX14*		7015	
ERP44			2794	*SLC35A4*		7040			*RAD50*		4026	
FKTN			2794	*RAD50*			4030		*ANGPTL4*		4025	
DEGS1			7026	*FBXO32*		2804	2805	2806	*DNAJC24*		4025	
EPHX1			7026	*ST3GAL1*	4	2804	2805	2806	*EVI5L*		4025	7016
PPP1R12B		7012	7026	*PTPN21*		357			*FAR1*	2	4025	7016
TNNT2	10	7012	7026	*VASH1*	7	357			*KCNN1*		4025	7016
ZBTB18		7012	7026	*ADD1*			7035		*MAP2K7*		4025	7016
EPHX1		7012		*HTT*	8		7035		*NACC1*		4025	7016
DEGS1		7012		*TADA2B*			7035		*ANGPTL4*			7016
STK24	11	7027		*ZFYVE28*			7035		*ISG12(A)*	7	7029	
ALDH18A1		2822	2824	*FOXO1*	11	7020	7023	7042	*SETD3*		7029	
ANKRD2		2822	2824	*CAMKK1*		7021	7036		*DDX58*		7031	
ARV1		2822	2824	*CAMTA2*		7021	7036		*DEGS1*		7034	
HIRA		2822	2824	*CHD3*	12	7021	7036		*EPHX1*		7034	
LYST		2822	2824	*KIF1C*		7021	7036		*ITGA8*		7031	
MTR		2822	2824	*MYH3*		7021	7036		*KIAA1462*	10	7031	
MYO18B	14	2822	2824	*MYO1C*		7021	7036		*PPP1R12B*		7034	
PTK2B		2822	2824	*ALDH18A1*		2823			*TNNT2*		7034	
NAA25		2822	2824	*ANKRD2*		2823			*TOPORS*		7031	
RSRC2		2822	2824	*SCARA5*		2823			*UBAP1*		7031	
SCARA5		2822	2824	*PTK2B*		2823			*ZBTB18*		7034	
WSB2		2822	2824	*C10orf10*		2823	4045		*FOXO1*	11	7030	

SDS		2822	2824	
IFIT1		2822	2824	
IFIT2		2822	2824	
IFIT3		2822	2824	4022
IFIT5		2822	2824	4022
JMJD1C		2822	2824	4022
P4HA1		2822	2824	4022
RUFY2		2822	2824	4022
SLC16A12		2822	2824	4022
C10orf10		2822	2824	4022
C14H10orf116		2822	2824	4022
BGN				4023
CD99L2	X			4023

C14H10orf116		2823	4045	
IFIT1		2823	4045	
IFIT2		2823	4045	
IFIT3		2823	4045	
IFIT5		2823	4045	
P4HA1		2823	4045	
SLC16A12		2823	4045	
RUFY2	14	2823	4045	7024
JMJD1C		2823		7024
LYST		2823		7024
MTR		2823		7024
SDS		2823		7024
HIRA		2823		7024
ARV1		2823		7024
WSB2		2823		7024
RSRC2		2823		7024
MYO18B		2823		7024
NAA25		2823		7024

ATXN7L3		7033
THRA	12	7033
PAXBP1	13	7032

Table S3. Localization of differentially expressed genes in QTL regions associated with diameter of all fiber type.

Type I muscle fibers				Type IIB muscle fibers			Type IIA muscle fibers			
**Gene**	**SSC**	**QTL ID**		**Gene**	**SSC**	**QTL ID**	**Gene**	**SSC**	**QTL ID**	
CCDC88C	7	2812		*CDO1*	2	2797	*ERP44*	1	2795	
PTPN21		2812		*CHD1*		2797	*FKTN*		2795	
VASH1		2812		*DMXL1*		2797	*TMEM38B*		2795	
DMTF1	9	2813		*F2RL2*		2797	*CDO1*	2	2796	
FAM185A		2813		*FCHO2*		2797	*CHD1*		2796	
SGCE		2813		*LVRN*		2797	*DMXL1*		2796	
CAMKK1	12	2819	2821	*PAPD4*		2797	*F2RL2*		2796	
CAMTA2		2819	2821	*POLR3G*		2797	*FCHO2*		2796	
CHD3		2819	2821	*RIOK2*		2797	*LVRN*		2796	
DYNLL2		2819	2821	*ANGPTL1*	9	7017	*PAPD4*		2796	
FLOT2		2819	2821	*C1orf27*		7017	*POLR3G*		2796	
KIF1C		2819	2821	*ZRANB1*	14	2827	*RIOK2*		2796	
MYH3		2819	2821	*FAM53B*		2827	*BIRC6*	3	7037	
MYO1C		2819	2821	*HIRA*		7018	*MAT2A*		7037	
TMEM100		2819	2821	*JMJD1C*		7018	*SLC9A2*		7037	
DPP4	15	2832		*MTR*		7018	*THUMPD2*		7037	
FKBP7		2832		*MYO18B*		7018	*TTL*		7037	
GPR155		2832		*NAA25*		7018	*ADORA3*	4	2807	2810
HNRNPA3		2832		*RSRC2*		7018	*ARHGEF11*		2807	2810
NABP1		2832		*RUFY2*		7018	*DENND4B*		2807	2810
NOSTRIN		2832		*SDS*		7018	*SLC19A2*		2807	2810
PRPF40A		2832		*WSB2*		7018	*SMG5*		2807	2810
SARAF		2832		*ARV1*		7018	*ST7L*		2807	2810
SF3B1		2832		*DPP4*	15	2833	*PTPN21*	7	358	
PIGA	X	7014		*FKBP7*		2833	*VASH1*		358	
RAB9A		7014		*GPR155*		2833	*FAM53B*	14	2828	
				HNRNPA3		2833	*ZRANB1*		2828	
				NABP1		2833	*ARV1*		7038	
				NOSTRIN		2833	*HIRA*		7038	
				PRPF40A		2833	*JMJD1C*		7038	
				SARAF		2833	*LYST*		7038	
				SF3B1		2833	*MTR*		7038	
				PIGA	X	7019	*MYO18B*		7038	
				RAB9A		7019	*NAA25*		7038	
							RSRC2		7038	
							RUFY2		7038	
							SDS		7038	
							WSB2		7038	
